# Interplay of multiple pathways and activity-dependent rules in STDP

**DOI:** 10.1371/journal.pcbi.1006184

**Published:** 2018-08-14

**Authors:** Gaëtan Vignoud, Laurent Venance, Jonathan D. Touboul

**Affiliations:** 1 Center for Interdisciplinary Research in Biology (CIRB) - Collège de France (CNRS UMR 7241, INSERM U1050), 11 Place Marcelin Berthelot, 75005 Paris, France; 2 Department of Mathematics and Volen National Center for Complex Systems, Brandeis University, Waltham, Massachusetts, United States of America; University of Pittsburgh, UNITED STATES

## Abstract

Hebbian plasticity describes a basic mechanism for synaptic plasticity whereby synaptic weights evolve depending on the relative timing of paired activity of the pre- and postsynaptic neurons. Spike-timing-dependent plasticity (STDP) constitutes a central experimental and theoretical synaptic Hebbian learning rule. Various mechanisms, mostly calcium-based, account for the induction and maintenance of STDP. Classically STDP is assumed to gradually emerge in a monotonic way as the number of pairings increases. However, non-monotonic STDP accounting for fast associative learning led us to challenge this monotonicity hypothesis and explore how the existence of multiple plasticity pathways affects the dynamical establishment of plasticity. To account for distinct forms of STDP emerging from increasing numbers of pairings and the variety of signaling pathways involved, we developed a general class of simple mathematical models of plasticity based on calcium transients and accommodating various calcium-based plasticity mechanisms. These mechanisms can either compete or cooperate for the establishment of long-term potentiation (LTP) and depression (LTD), that emerge depending on past calcium activity. Our model reproduces accurately the striatal STDP that involves endocannabinoid and NMDAR signaling pathways. Moreover, we predict how stimulus frequency alters plasticity, and how triplet rules are affected by the number of pairings. We further investigate the general model with an arbitrary number of pathways and show that depending on those pathways and their properties, a variety of plasticities may emerge upon variation of the number and/or the frequency of pairings, even when the outcome after large numbers of pairings is identical. These findings, built upon a biologically realistic example and generalized to other applications, argue that in order to fully describe synaptic plasticity it is not sufficient to record STDP curves at fixed pairing numbers and frequencies. In fact, considering the whole spectrum of activity-dependent parameters could have a great impact on the description of plasticity, and a better understanding of the engram.

## Author summary

The brain’s capacity to treat information, learn and store memory relies on synaptic connectivity patterns, which are altered through synaptic plasticity mechanisms. Experimentally, such plasticities were evidenced through protocols involving numerous repetitive stimulations of a given synapse, and were shown to be supported by multiple pathways. Using a simple biologically grounded mathematical model, we show how activation timescales and inactivation levels of each pathway interact and alter plasticity in an intricate manner as stimuli are presented. Building upon data from the synapse between cortex and striatum, we show that synaptic changes may revert or re-emerge as stimuli are presented, and predict specific responses to changes in stimulus frequency or to distinct simulation patterns. Our general model shows that a given plasticity profile emerging in response to a repetitive stimulation protocol can unfold into various scenarii upon variations of the number of stimulus presentations or patterns, which tightly depends on the underlying activated pathways. Altogether, these results argue that in order to better understand learning and memory, single plasticity responses obtained through intensive stimulations do not reveal the complexity of the responses for smaller number of presentations, which may have a strong impact in fast learning of stimuli with low numbers of presentations.

## Introduction

Synaptic plasticity, one of the paramount biological mechanism supporting learning and memory in the brain, has been the object of a wide literature spanning from experimental works [1–3] to computational investigations [4–6]. In 1949, Donald Hebb’s pioneering work postulated that long-term modifications of the synaptic efficacy can be induced in response to patterns of activity of the pre- and postsynaptic neurons [7]. Since then, many experimental studies have confirmed and extended Hebb’s postulate and have highlighted the complexity of the signaling pathways and their neuromodulation leading to synaptic efficacy changes in response to pre- and postsynaptic activity patterns [1, 2, 8, 9]. Numerous mathematical models were also developed to emulate this diversity and infer their computational capabilities [4–6].

Spike-timing-dependent plasticity (STDP) is a biological phenomenon of activity-dependent change in synaptic connectivity that is viewed as a synaptic Hebbian learning rule. STDP has been widely studied in the last two decades and experimentally observed at many synapses in various forms, and those were classified depending on the manner in which they implement Hebb’s postulate [8, 9].

STDP is assessed experimentally through repetitive paired activations of the pre- and postsynaptic sites with a prescribed timing that is denoted in this paper Δ*t*. By convention, we consider Δ*t* < 0 when the postsynaptic stimulation occurs before the paired presynaptic one (post-pre pairings), and Δ*t* > 0 when the presynaptic stimulation occurs before the postsynaptic one (pre-post pairings). Classically, the same paired stimulation is repeated between 80 and 150 times at a constant frequency (between 0.1 and 5 Hz) [8–10]. In many cases, these pairing patterns induce long-term plasticity exhibiting various polarities (increase or decrease of the synaptic weight as a function of the sign of Δ*t*) or magnitudes. In the vast majority, the expression of STDP is restricted to a narrow interval of values for Δ*t*; thus, when pre- and postsynaptic activities are separated by a large Δ*t* (|Δ*t*| > 50 ms in most of the cases), no long-term plasticity is observed [11, 12].

In this study, we term *Hebbian STDP* the plasticities whereby sequences of presentations of a presynaptic spike followed by a postsynaptic spike lead to Long-Term Potentiation (LTP) when repeated a specific number of times (denoted *N*_*Pairings*_) at a certain frequency, whereas reverse sequences induce Long-Term Depression (LTD). Hebbian STDP was reported in various structures such as the hippocampus [11, 13–15], the cerebral cortex [12, 16–19] and the striatum [20–23]. Conversely, we will term here (bidirectional) *anti-Hebbian* STDP, the forms of STDP exhibiting a reverse polarity when compared to the aforementioned Hebbian STDP: causal pre-post pairings induce LTD and anti-causal post-pre pairings induce LTP. Bidirectional anti-Hebbian STDP was also observed, for instance in the striatum [24–28], in the somatosensory cortex [29] or in the cerebellum-like structure of the electrical fish [30]. Unidirectional anti-Hebbian STDP, i.e. LTD for both post-pre and pre-post pairings, is another main form of STDP observed in the neocortex [31, 32], the dorsal cochlear nucleus [33], the cerebellum [34, 35] and the hippocampus [36]. We underline that different definitions of (anti-)Hebbian STDP were used in the literature; the present study follows the terminology of early experimental studies [11, 12], or Figure 2 of the review [8], but differs, e.g., from the definitions used in [37].

These plasticities were shown to be dependent upon the parameters of the stimulation beyond spike-timing: for instance, varying the frequency at which pairings are presented or the total number of pairings, presenting distinct spike patterns (triplets, single spike, theta bursts, …) [17, 38–41] or changing neuromodulatory tones [21] may lead to distinct forms of STDP.

Despite the existence of multiples forms of STDP [8, 9], all of them have in common the crucial role played by the calcium transients in the pre- and postsynaptic compartments for the induction and maintenance of plasticity. Postsynaptic calcium influxes through NDMA receptors (NMDAR) and voltage-sensitive calcium channels have been demonstrated to be key factors governing plasticity expression and polarity [10]. Regarding Hebbian plasticity, calcium-dependent mechanisms act as coincidence detectors, essential to implement any type of STDP. In addition, distinct signaling pathways appear to be involved, namely (i) calcium triggering downstream cascades modulating calcium/calmodulin-dependent kinase II (CaMKII) [42] which ultimately regulates the gene expression and/or (ii) the endocannabinoid (eCB) system, whose synthesis and release is calcium-dependent, acting retrogradely on the presynaptic element [43–45]. Importantly, both of these pathways are able to trigger LTP or LTD depending on the spatio-temporal kinetics of the calcium [19, 40, 46]. Calcium dynamics thus constitute a key factor in synaptic plasticity induction and in selecting plasticity forms. Accordingly, numerous mathematical models were based on calcium transients and described various forms of STDP [5]. In particular, Graupner and Brunel [47] proposed simple calcium-based models able to account for a wide range of experimental observations on synaptic plasticity.

However, while calcium-based models succeed in reproducing the results of the “classical” STDP (∼ 100 pairings), they do not take into account the dynamics of the establishment of plasticity and the variety of timescales involved in plasticity induction. Indeed, in computational neuroscience, it is implicitly admitted that the synapse gradually amplifies synaptic changes as the number of stimulus presentation increases to reach the final plasticity profiles. However, plasticity occurs at vastly distinct timescales and protocols based on one hundred trials (i.e., pairings), as classically performed in STDP experiments, only reveal an extreme steady state outcome. Actually, a dozen of trials can be sufficient to induce plasticity, if not less in the case of associative memory [39, 40, 48, 49]. Importantly, it was recently shown that depending on the number of pairings, STDP on the cortex-to-striatum synapses (cortico-striatal STDP) exhibits three distinct forms of plasticity: an NMDAR-mediated LTP and an eCB-mediated LTD for 100 post-pre and pre-post pairings, respectively [20, 21, 24, 25, 27], and an eCB-mediated LTP for 5-15 post-pre pairings [39, 40]. Note that at cortico-striatal synapses, GABA operates as a Hebbian/anti-Hebbian switch [27, 28] and that without the blockade of GABA transmission, an anti-Hebbian STDP is induced as observed *in vivo* [26]. These phenomena were reproduced in a biophysical model of the cortico-striatal synapse accounting for receptors activation dynamics (36 equations and 150 parameters) [40]. However, no simple phenomenological model reproduces these phenomena, and in particular models of plasticity based on the calcium hypothesis fail to reproduce such complex dynamical emergence of plasticity.

Here, we propose a new model built upon the calcium hypothesis and taking into account the existence of multiple signaling pathways at a given synapse that may be activated at distinct calcium levels. We instantiate one model to fit the data from cortico-striatal STDP [39, 40], and show that the model accurately reproduces the experiments on the dependence of STDP on both the number and frequency of pairings. We use this model to predict the response of the system as the stimulus frequency and number of presentations are varied, and extend the model to show how triplet rules will depend on the number of stimulus presentations. Our model goes beyond the case of the cortico-striatal synapse with two signaling pathways, and we further explore the diversity and limited range of dynamical plasticity establishments that can be unfolded from classical Hebbian STDP. In the face of this diversity, we further propose experimentally implementable protocols to differentiate those scenarii. This study thus sheds a new light on the interplay of multiple signaling pathways at single synapses and how this multiplicity endows the synapse with the capacity of encoding multiple STDP profiles depending on the number and frequency of stimulus presentation, and argues that experiments based on stereotypical stimulus presentations are not sufficient to finely account for the complexity of plasticity, even in widely studied synapses.

## Results

### A generalized model for STDP with multiple calcium-based mechanisms

We developed a general calcium-based model of the synapse allowing to take into account the presence of multiple pathways and past activity in the establishment of plasticity. Our developments build upon the Graupner and Brunel model [47], and extend it by (i) introducing multiple plasticity pathways, and (ii) taking into account the fact that receptor activation thresholds may depend on past activity. We provide here the details of the models and the emergent changes in synaptic weight, as well as a theoretical formula thereof.

#### A heterogeneous synapse model

Motivated by the variety of biological situations in which multiple pathways take part in STDP, such as the cortico-striatal [20, 24–28, 39] or neocortical [16–19, 33, 50, 51] synapses, we introduced a new simplified calcium-based model reproducing the dynamics of the establishment of STDP in these situations. The calcium-based model for STDP introduced in [47] describes changes in the individual synaptic efficacy as a one-dimensional variable *ρ*, function of postsynaptic calcium transients. This variable can be stabilized into one of two states, potentiated or depressed, depending on the activation of potentiation and depression mechanisms triggered when the instantaneous calcium concentration exceeds specific thresholds. At long timescales and for experiments involving large numbers of stimulus presentations, this model is able to accurately reproduce the plasticity rules observed experimentally. However, this model was not designed to reproduce the precise changes in plasticity for various numbers of stimulus presentations, and does not distinguish the respective impact of distinct pathways. Moreover, because it considers a unique plasticity pathway, the model generates progressive (monotonic) plasticity inductions as the number of pairings increases (see S1 Fig for an example of monotonic Hebbian plasticity). To account for non-monotonic plasticity inductions, we define here a new model based on the assumptions of [47], conserving the same calcium dynamics, but expanded to incorporate multiple signaling pathways and history dependence.

When multiple signaling pathways contribute to the establishment of plasticity, calcium activation thresholds are distinct in each pathway, and thus the contribution of each pathway may be distributed over time. Moreover, the temporal dynamics of the establishment of plasticity require refining the model and dropping the assumption of [47] that only instantaneous calcium transients play a role. Indeed, although the dynamics of uptake and release of calcium are fast, LTP and LTD activation thresholds depend upon the past activity of the cell. This is due to a variety of phenomena, including the limited resource of cytoplasmic calcium in the vicinity of the synapse (resulting in the decrease of calcium spikes amplitudes with repeated stimulation), pre- and postsynaptic receptor desensitization and saturation mechanisms. To take into account these phenomena, we expanded the model introduced in [47] to the plasticity of a heterogeneous synapse with *P* plasticity pathways whose state is given by *P* synaptic efficacies (*ρ*_*α*_)_*α*∈{1⋯*P*}_. We conserve, at the level of each individual pathway, the assumption made in [47] and experimentally motivated [52], that the synaptic efficacies are in one of the two states: potentiated or depressed, depending on the calcium transients following pre- and postsynaptic stimulations triggering biochemical cascades leading either to LTP or LTD [53, 54]. The different signaling pathways are assumed to be independent functions of (the same) postsynaptic calcium concentration *c*(*t*), and individual synaptic efficacies follow the system of stochastic differential equations:

$$\begin{array}{c} \tau \frac{d\rho_{\alpha }}{dt}=-\rho_{\alpha }\left(1-\rho_{\alpha }\right)\left(\rho_{*}-\rho_{\alpha }\right)+\gamma_{\alpha }^{p}\left(1-\rho_{\alpha }\right)\Theta \left[c\left(t\right)-\theta_{\alpha }^{p}\left({\tilde{c}}_{t}\right)\right]\\ -\gamma_{\alpha }^{d}\rho_{\alpha }\Theta [c(t)-\theta_{\alpha }^{d}({\tilde{c}}_{t})]+{\text{Noise}}_{\alpha }\left(t\right), \end{array}$$ (1)

where ${\tilde{c}}_{t}≔{\left(c\left(s\right)\right)}_{0\leq s\leq t}$ is the past values of calcium concentration up to time *t*. The calcium concentration is identical to the model of [47]. In detail, calcium concentration relaxes exponentially to its equilibrium value (here chosen equal to 0 without loss of generality) with a time constant *τ*_*Ca*_ in the absence of spikes. Each pre- or postsynaptic spike (occurring at times denoted respectively $t_{i}^{pre}$ and $t_{j}^{post}$), evokes a calcium peak with amplitude *C*_*pre*_ and *C*_*post*_ respectively:

$$\begin{array}{c} \frac{dc(t)}{dt}=-\frac{1}{\tau_{Ca}}c\left(t\right)+C_{pre}\sum_{t_{i}^{pre}<t}\delta \left(t-t_{i}^{pre}-D\right)+C_{post}\sum_{t_{j}^{post}<t}\delta \left(t-t_{j}^{post}\right), \end{array}$$ (2)

where *D* models the relative delay between calcium influx in response to a post- and a presynaptic spike (note that this delay can be positive or negative depending on the respective properties of pre- and postsynaptic calcium responses).

In Eq (1), *τ* denotes the time constant of the synapse evolution (on the order of a few minutes and slower than the calcium dynamics); it is assumed to be identical for each pathway. The bistable nature of each synaptic efficacy is incorporated in the model through the nonlinear (cubic) term: the potentiated corresponds to *ρ*_*α*_ = 1 and the depressed state to *ρ*_*α*_ = 0, and the synaptic efficacy converges to one of these two states after a stimulation protocol depending on whether the stimulus has brought the individual efficacy, respectively, higher or lower than a value ρ_*_.

The individual synaptic efficacy varies depending on the calcium concentration through LTP and LTD mechanisms that are activated when the calcium concentration exceeds specific thresholds $\theta_{\alpha }^{p}$ and $\theta_{\alpha }^{d}$ intrinsic to each synaptic pathway and that depend on past calcium concentration (see next section). In detail, as soon as the calcium trace reaches $\theta_{\alpha }^{p}$ (resp., $\theta_{\alpha }^{d}$), the rate of variation of the individual synaptic efficacy increases of intensity $\gamma_{\alpha }^{p}$ (resp., decreases of $\gamma_{\alpha }^{d}$). This phenomenon is modeled by the presence of the Heaviside step-function Θ(*x*) = 0 if *x* < 0 or Θ(*x*) = 1 otherwise. As noted above, the thresholds for LTP and LTD depend on the calcium concentration due to a variety of homeostatic or biochemical (deactivation) phenomena.

A noise term only active during the phases where LTP and LTD occur, completes the model. These fluctuations are intrinsic to each pathway and given by a Gaussian white noise with diffusion coefficient:

$$\begin{array}{c} {\text{Noise}}_{\alpha }\left(t\right)=\sigma \sqrt{\tau }\sqrt{\left(\Theta [c(t)-\theta_{\alpha }^{p}({\tilde{c}}_{t})]+\Theta [c(t)-\theta_{\alpha }^{d}({\tilde{c}}_{t})]\right)}\;\eta_{\alpha }\left(t\right) \end{array}$$ (3)

with *σ* the noise intensity and (*η*_*α*_(*t*))_*α*∈{1⋯*P*}_ a collection of *P* independent Gaussian white noise processes.

**Fig 1:**
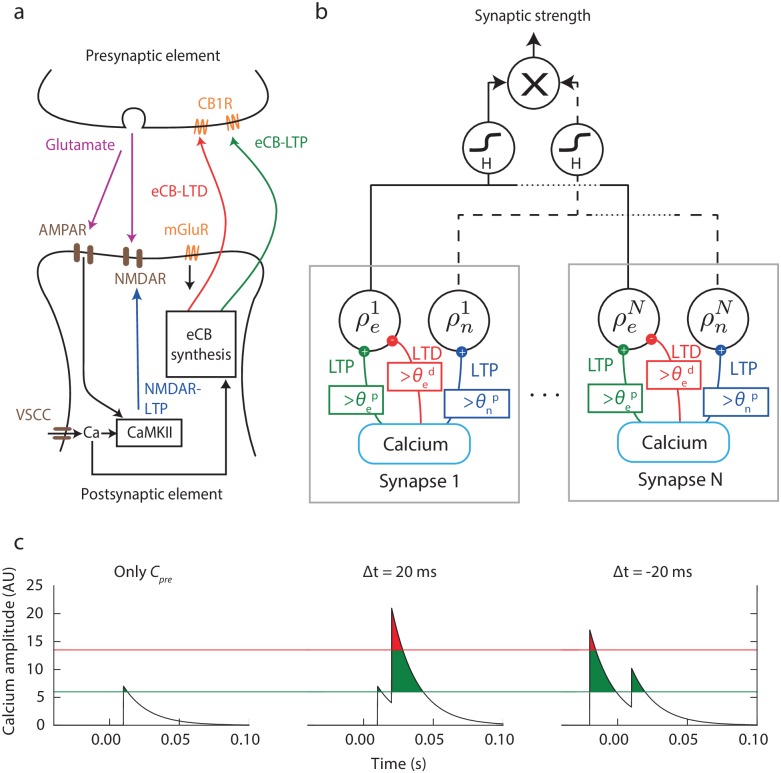
Model for the cortico-striatal STDP. **(a)** Schematic representation of the cortico-striatal synapse: three independent mechanisms of plasticity can be induced in response to calcium influx: eCB-LTD, eCB-LTP and NMDAR-LTP. The dynamics of the NMDAR pathway rely on released glutamate activating NMDAR and AMPA receptors (AMPAR), ultimately acting on calcium-cadmodulin released glutamate activating NMDAR and AMPA receptors (AMPAR), ultimately acting on calcium-cadmodulin voltage-sensitive calcium channels (VSCC). The eCB synthesis is under the control of metabotropic glutamate receptors (mGluR) and calcium concentration, and acts retrogradely on CB1 receptors (CB1R) on the presynaptic element. **(b)** Block diagrams of the reduced calcium-based model proposed where each block corresponds to a different individual synapse whose efficiency splits into individual efficacy of NMDAR and eCB pathways. The proportion of synapses changing states are then computed and the sigmoid function *H* is then applied. The macroscopic change is the product of the contribution of each individual pathway. **(c)** Calcium spikes and plasticity thresholds for different STDP protocols (see parameters in Table 1, thresholds are those associated with the eCB pathway, red for LTD and green for LTP).

Heuristically, this model states that an individual synaptic efficacy switches from a potentiated to a depressed state as soon as the LTD terms exceed, for a sufficient duration, the potentiation terms, so that the individual synaptic efficacy eventually crosses the critical value ρ_*_. These switches strongly depend on calcium concentration, in turn depending on the precise timing and order of pre- and postsynaptic traces, see Fig 1(c). In response to a paired activity on either side of the synapse, each pairing can trigger LTP and/or LTD, or have no effect, depending on whether the calcium transients exceed LTP/LTD thresholds. The repetition of this pairing at a certain frequency and a fixed number of times (on the order of 100) reinforces these effects, summing up these elementary synaptic changes and possibly leading to a switch between the potentiated and the depressed states or conversely.

#### Activation thresholds

The assumption of constant activation thresholds for LTP and LTD in [47], while being sufficient to reproduce a variety of plasticity profiles, cannot account for the variability observed as the number of pairings varied [17, 39, 40, 55]. This dependence on past activity likely is a multifarious phenomenon, relying for instance upon homeostasic mechanisms and reversible (but durable) changes in the properties of receptors after sustained stimulation. Accounting for these phenomena require considering threshold depending on the past cell activity. To incorporate these effects in our model, we assume that LTP and LTD inactivate when the cumulative calcium concentration exceeds a given threshold. We will consider two such models:

- the exponential threshold model in which the inactivation occurs in a finite (but brief) time of order *ϵ*:
  $$\begin{array}{c} \theta_{\alpha }^{x}\left({\left(c\left(s\right)\right)}_{0\leq s\leq t}\right)=\theta_{\alpha ,0}^{x}+\text{exp}\left(\frac{1}{\epsilon }\left(\int_{0}^{t}c\left(s\right)ds-\mu_{\alpha }^{x}\right)\right) \end{array}$$
  for each plasticity pathway *α* ∈ {1, ⋯, *P*}, with *x* = *p* or *d*. In this equation, $\mu_{\alpha }^{x}$ denotes the calcium levels beyond which receptors are no more responsive, thus silencing the LTP or LTD of mechanism *α*.
- the piecewise constant threshold case, corresponding to the limit of the exponential threshold model when *ϵ* is very small compared to the observation time:
  $$\begin{array}{c} \theta_{\alpha }^{x}\left({\left(c\left(s\right)\right)}_{0\leq s\leq t}\right)=\{\begin{array}{ll} \theta_{\alpha ,0}^{x} & \text{if}\;\int_{0}^{t}c\left(s\right)ds-\mu_{\alpha }^{x}<0\\ +\infty & \text{otherwise} \end{array} \end{array}$$
  The piecewise constant approximation will be particularly useful for our analytical computation of the solutions of the system below.

This history-dependent thresholds is evocative of the early works on homeostasis in a learning rule for the visual cortex, in particular by Bienenstock, Cooper and Munro [56, 57]. This BCM model introduced such a dependence at the level of a given cell’s firing rate through a modulation of F-I curves upon the average firing rate received. The history-dependent thresholds used in our model thus describe distinct phenomena that are more local (a single synapse vs neural assemblies), and probably faster (desenzitisation or homeostasis via calcium concentrations vs slower modulation of firing rates).

#### Computation of the macroscopic synaptic strength

The model described in the previous sections represents the dynamics of one given synapse subject to multiple plasticity pathways. Biological experiments generally stimulate a large number of synapses, on the order of hundreds to thousands, each being subject to its own intrinsic fluctuations. To account for the emerging change in synaptic weight resulting from these interactions, we considered a system composed of *N* synapses described as above [47], whose initial state is uniformly distributed between the potentiated and depressed steady states, and since they respond to the same stimulations, they perceive the same calcium concentration. The effective change in synaptic transmission is computed as the proportion of synapses that changed state during the protocol, and this quantity is directly compared to electrophysiological experimental measurements.

In detail, the *macroscopic synaptic strength* modification after a stimulation paradigm is related to the collection of individual synaptic efficacies $(\rho_{\alpha }^{i}{)}_{i=1\cdots N}$ through the proportion of synapses that got potentiated $U_{\alpha }={\mathbb{P}}_{\alpha }(0\to 1|p_{0})$ for pathway *α*, and the proportion of depressed synapses $D_{\alpha }={\mathbb{P}}_{\alpha }(1\to 0|p_{0})$, both depending on the proportion *p*_0_ of synapses in the depressed state prior to the stimulation. As this proportion is not controllable experimentally, we propose here a new method to extract the synaptic strength whose fit with experimental data does not require to make an assumption of asymmetry of the initial state. To this end, we assume that the change in the macroscopic synaptic strength is a non-decreasing function *H* of the ratio *U*_*α*_/*D*_*α*_ (conserving the relevant monotonicity in those two variables), with *H* being such that:

- *H*(1) = 1: if the proportion of potentiated synapses is equal to the proportion of depressed synapses, the macroscopic synaptic efficacy remains as before;
- *H*(0) = LTD^⋆^: the maximal depression value, observed experimentally, reached when no individual efficacy is potentiated (*U*_*α*_ = 0, *D*_*α*_ > 0). Indeed, in that case, regardless of the initial proportion of potentiated synapses, all synapses will in the long run get depressed and remain in that state for subsequent times.
- *H*(∞) = LTP^⋆^ the maximal potentiation, observed experimentally, reached when no depression occurs (*D*_*α*_ = 0, *U*_*α*_ > 0).

In our model, we consider the following sigmoidal function:

$$\begin{array}{c} H(\frac{U}{D})=a+\frac{b}{1+e^{-s(\frac{U}{D}-d)}} \end{array}$$ (4)

where *s* is a slope parameter and the only free parameter to be fitted to the data once the values LTP^⋆^ and LTD^⋆^ are extracted. Indeed, the above conditions impose the following formulae for the coefficients:

$$\begin{array}{c} \left\{ \begin{array}{c} d=\frac{1}{s}\log(\frac{\Delta -e^{s}}{1-\Delta }),\\ b=\frac{{\text{LTP}}^{⋆}-{\text{LTD}}^{⋆}}{1-\frac{1}{1+e^{s\;d}}},\\ a={\text{LTP}}^{⋆}-b \end{array}\right. \end{array}$$

with Δ = (LTP^⋆^ − LTD^⋆^)/(LTP^⋆^ − 1).

When experiments allow activating the distinct pathways separately, the parameters of the map *H* can be specified for each pathway *α* ∈ {1⋯*P*}; the associated transform *H*_*α*_ is derived specifically evaluating the values of *LTP*^⋆^, *LTD*^⋆^ and *s* associated to that pathway. Since in most cases we do not have access to this data, we shall consider here that all pathways have an identical map *H* whose parameters are evaluated on the resulting dynamics. Given a postsynaptic calcium trace, the efficacies from different pathways are mutually independent, since they are driven by independent random fluctuations (see Eq (1)). Therefore, the resulting macroscopic synaptic strength modification due to all pathways is given by:

$$\begin{array}{c} \text{Total}\;\text{change}\;\text{in}\;\text{macroscopic}\;\text{synaptic}\;\text{strength}=\prod_{\alpha =1}^{P}H_{\alpha }(\frac{U_{\alpha }}{D_{\alpha }}) \end{array}$$ (5)

In our results, we will compare this variable to the change in macroscopic synaptic strength estimated experimentally as the relative change in EPSC size after the stimulation protocol is applied.

#### Mean-field approximation and theoretical solution

The nonlinear dynamical system described above is well-posed and its solutions can be computed numerically using simulations based on the Euler-Maruyama numerical scheme. In order to finely understand the structure of the system and obtain extensive and rapid simulations, we derive here an approximate explicit analytical solution of the system, valid under the assumptions that:

1. The number of synapses is large (*N* ≫ 1): in that case, since elementary efficiencies are independent realizations of the same process, the proportion of synapses getting potentiated or depressed is well approximated by the probability that one given efficiency performs the associated switch, as a result of the law of large numbers. Moreover, the central limit theorem implies that this approximation is accurate to order $O(1/\sqrt{N})$;
2. Thresholds are piecewise constant, and only depend on past activity through the total number of past pre- and postsynaptic spikes. This hypothesis is relevant here because the integral calcium does not depend, in first approximation, on the precise timing between the pre- and postsynaptic spikes;
3. a single calcium transient induces a small change in individual synaptic efficacy during one pairing and the cubic term can be neglected.

Under these assumptions, the stochastic equation Eq (1) reduces to a linear Eq (7), whose solution is given by an Ornstein-Uhlenbeck process with switching coefficients (varying as a step function). These processes can be fully characterized analytically and provide a very efficient way to compute synaptic changes resulting from a stimulation protocol, as made explicit in the Methods section. We note that this approximation only models transient dynamics occurring during the protocol; after the end of stimulation, the calcium concentration will decay and the dynamics become deterministic (the standard deviation of the noise term is equal to 0 as soon as *c* is less than the potentiation and depression thresholds, see Eq (3)). Because the dynamics becomes deterministic and bistable after the protocol, the probabilities *U* and *D* computed at the end of the protocol are the same as the steady state probabilities.

### NMDAR- and endocannabinoid-dependent plasticity at cortico-striatal synapses

The model we built in the previous section is general and is thus able to reproduce a variety of synapses and plasticity mechanisms relying on calcium dynamics. We study in this section the case of STDP at the cortico-striatal synapse, which was studied experimentally with varying *N*_*pairings*_ [39, 40]. In these contributions, it was shown that STDP at the cortico-striatal synapse relied both on NMDAR and endocannabinoid pathways (see Fig 1(a)), and that synaptic changes after paired pre- and postsynaptic spikes not only depended on the timing between the pre- and postsynaptic spikes, but also varied with the number and the frequency of the pairings presented. Namely, it was shown that for pre-post pairings (0 < Δ*t* < +40 ms), an eCB-LTD progressively appeared as the number of pairings was increased, while for post-pre pairings (−30 ms < Δ*t* < 0 ms), a biphasic STDP emerged with an eCB-LTP for a low number of pairings (5 − 15 pairings), an absence of plasticity between 25 and 50 pairings, leaving room for NMDAR-LTP at higher numbers of pairings (≥ 75 pairings). A schematic representation of the biological pathways involved is provided in Fig 1(a) together with the biophysical mechanisms and proteins cascades (described in more detail in [40]).

A minimal model of cortico-striatal plasticity thus requires taking into account two different and independent calcium-dependent pathways (*P* = 2), an eCB-dependent mechanism (*α* = *e*) which induces both LTP or LTD depending on the specific timing Δ*t* of the pairings, and an NMDAR-dependent (*α* = *n*) associated to LTP only. This yields to the following system of stochastic differential equations (see schematic diagram in Fig 1(b)):

$$\{\begin{array}{ll} \tau \frac{d\rho_{e}}{dt}= & -\rho_{e}\left(1-\rho_{e}\right)\left(\rho_{*}-\rho_{e}\right)+\gamma_{e}^{p}\left(1-\rho_{e}\right)\Theta \left[c\left(t\right)-\theta_{e}^{p}\left({\tilde{c}}_{t}\right)\right]\\ & -\gamma_{e}^{d}\rho_{e}\Theta \left[c\left(t\right)-\theta_{e}^{d}\left({\tilde{c}}_{t}\right)\right]+{\text{Noise}}_{e}\left(t\right)\\ \tau \frac{d\rho_{n}}{dt}= & -\rho_{n}\left(1-\rho_{n}\right)\left(\rho_{*}-\rho_{n}\right)\\ & +\gamma_{n}^{p}\left(1-\rho_{n}\right)\Theta \left[c\left(t\right)-\theta_{n}^{p}\left({\tilde{c}}_{t}\right)\right]+{\text{Noise}}_{n}\left(t\right) \end{array}$$ (6)

The complete synapse model is made of *N* ≫ 1 independent pairs $(\rho_{e}^{i},\rho_{n}^{i}{)}_{i=1\cdots N}$ satisfying Eq (6), and a synaptic change deduced from the proportion of synapses that switch from being potentiated to depressed or reciprocally, through the sigmoidal map *H* of Eq (4). As described above, in response to pre- and postsynaptic spike-timing (Δ*t* = *t*_*post*_ − *t*_*pre*_), the calcium dynamics *c* undergoes jumps followed by exponential relaxation as described in Eq (2), activating eCB-LTP, eCB-LTD and NMDAR-LTP as soon as *c* exceeds specific LTP or LTD thresholds (see Fig 1(c), where the LTP and LTD thresholds are represented by the green and red lines respectively) with both thresholds taken from the adjusted parameters of Table 1 for the eCB pathway. When only one presynaptic spike (thus without postsynaptic spike) is evoked, the calcium concentration amplitude exceeds the LTP threshold for a short amount of time, and remains below the level of LTD induction: repeating this protocol does not lead to significant plasticity. For a pre-post stimulation (Δ*t* > 0), the summation of the pre- and postsynaptic calcium spikes triggers both LTP and LTD. The same is valid for Δ*t* < 0, but the relative time spent above the LTP and LTD thresholds would be significantly different depending on Δ*t*, underlining the importance of the timing and order between the spikes in the resulting plasticity: in the example depicted in Fig 1(c) and parameters $\gamma_{\alpha }^{x}$ from Table 1, a pre-post stimulation yields LTD whereas a post-pre stimulation yields LTP, consistent with anti-Hebbian STDP at cortico-striatal synapses *ex vivo* [27, 28] in the absence of GABA_A_ receptor antagonist and *in vivo* [26].

**Table 1:** Default parameters.

Cortico-striatal STDP Figs 1–8, S2 and S3 and M1		Symmetric anti-Hebbian LTD Figs 9 and S4				Hebbian STDP from [47] S1 Fig		
ϵ = 1 for Figs 2 and S2		Scenario	1	2	3	Scenario	Asymmetric	Symmetric
*C*_*pre*_	7	*C*_*pre*_	7	7	7	*C*_*pre*_	1	2
*C*_*post*_	17.1	*C*_*post*_	7	15	15	*C*_*post*_	2	2
*τ*_*Ca*_	18 ms	*τ*_*Ca*_	17 ms	17 ms	17 ms	*τ*_*Ca*_	20 ms	20 ms
*D*	10 ms	*D*	0	10 ms	ms	*D*	13.7 ms	0
ρ_*_	0.5	ρ_*_	0.5	0.5	0.5	ρ_*_	0.5	0.5
*τ*	165	*τ*	280	280	280	*τ*	150	150
*σ*	1	*σ*	1	1	1	*σ*	2.8284	2.8284
LTP^⋆^	3,475	LTP^⋆^	4	4	4	LTP^⋆^	4	4
LTD^⋆^	0.55	LTD^⋆^	0.5	0.5	0.5	LTD^⋆^	0.5	0.5
*c*	0.7	*c*	0.5	0.5	0.5	*c*	0.5	0.5
$\theta_{e,0}^{p}$	6	$\theta_{A,0}^{p}$		6	11	$\theta_{A,0}^{p}$	1,3	1,3
$\gamma_{e}^{p}$	290	$\gamma_{A}^{p}$		430	420	$\gamma_{A}^{p}$	321.808	257.447
$\mu_{e}^{p}$	6	$\mu_{A}^{p}$		8	+∞	$\mu_{A}^{p}$	+∞	+∞
$\theta_{e,0}^{d}$	13.5	$\theta_{A,0}^{d}$	6	10	10	$\theta_{A,0}^{d}$	1	1
$\gamma_{e}^{d}$	250	$\gamma_{A}^{d}$	190	220	360	$\gamma_{A}^{d}$	200	160
$\mu_{e}^{d}$	+∞	$\mu_{A}^{d}$	+∞	29	+∞	$\mu_{A}^{d}$	+∞	+∞
$\theta_{n,0}^{p}$	5.8	$\theta_{B,0}^{d}$		5.8	14			
$\gamma_{n}^{p}$	50	$\gamma_{B}^{d}$		100	550			
$\mu_{n}^{p}$	32	$\mu_{B}^{d}$		25	25			
*N*	1000	*N*	1000	1000	1000	*N*	1000	1000
*N*_*iter*_	102000	*N*_*iter*_	204000	204000	204000	*N*_*iter*_	102000	102000

#### Dependence of the different thresholds on the calcium trace

To complete the definition of the model, we defined the three thresholds $\theta_{n}^{p}$, $\theta_{e}^{p}$ and $\theta_{e}^{d}$ and their variations as a function of the calcium integral. To this purpose, we have compared the model results to electrophysiological recordings obtained at cortico-striatal synapses [39, 40].

Based on the fact that eCB-LTP disappears around 25 pairings in cortico-striatal STDP, we defined the LTP activation threshold to switch from activated to inactivated at a value evaluated as $\mu_{e}^{p}=6$ which corresponds to 14 pairings (the LTP lingers for some time before disappearing). Homeostatic and saturation mechanisms take place to overcome unlimited potentiation, which was modeled considering, consistently with the electrophysiological data [39, 40], that NMDAR-LTP inactivates above a specific level of cumulated calcium, set to 74 pairings, corresponding to $\mu_{n}^{p}=32$. eCB-LTD was considered active during the whole STDP pairings. We did not add an additional homeostasis-and-saturation mechanism for eCB-LTD in our minimal model, to avoid introducing additional parameters and the risk of overfitting.

**Fig 2:**
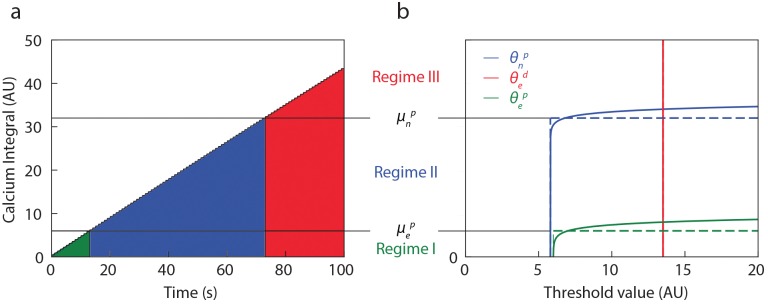
Activity-dependent thresholds. **(a)** Cumulative calcium concentration as a function of time for 100 pairings at 1 Hz (Δ*t* = 0). **(b)** Thresholds for eCB-LTP (green), eCB-LTD (red) and NMDAR-LTP (blue) as a function of the cumulative calcium concentration (dotted lines: piecewise constant model, solid lines: exponential thresholds, *ϵ* = 1). Three typical regimes of plasticity emerge depending on which pathway is activated (see text).

These thresholds thus define three distinct regimes of cumulated calcium concentration with specific active plasticities (see Fig 2):

- Regime I (0-14 pairings): both eCB-LTP, eCB-LTD and NMDAR-LTP are active,
- Regime II (15-74 pairings): eCB-LTD and NMDAR-LTP are active, eCB-LTP is inactive,
- Regime III (75+ pairings): only eCB-LTD is active.

#### Analytical solution for cortico-striatal STDP

**Fig 3:**
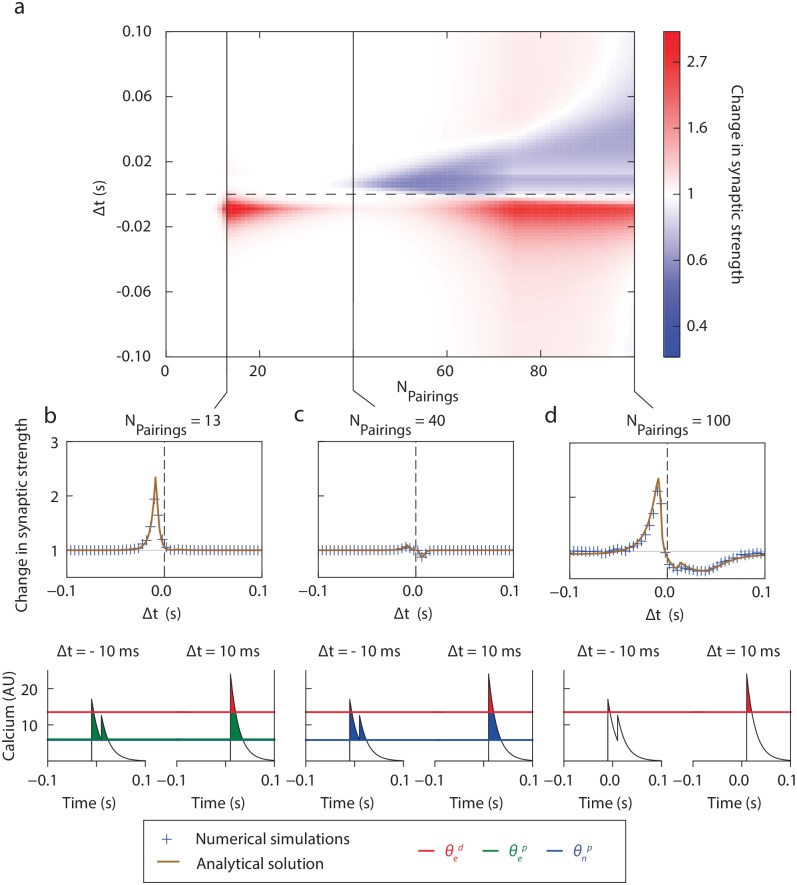
Cortico-striatal STDP and its dependence with pairing number. **(a)** Change in macroscopic synaptic strength as a function of the number of pairings *N*_*pairings*_ and spike timing Δ*t* computed with the analytical formula derived in the Methods section shows a non-monotonic LTP induction for Δ*t* < 0 and monotonic LTD establishment of Δ*t* > 0. The respective role of eCB and NMDAR pathways is investigated in Fig 5(b)–5(d) Sample STDP profiles for 13 **(b)**, 40 **(c)** and 100 **(d)** pairings (top), with calcium traces associated with Δ*t* = −10 ms (left) and Δ*t* = +10 ms (right). Analytical solution (Eqs (16) and (17), brown line) and numerical simulations of Eq (6) (blue crosses) are both represented for comparison.

We fitted this model to the data of the cortico-striatal synapse. Because of the number of system parameters compared to the experimental points, we developed a multi-step fitting algorithm consistent with the underlying biological system. We fitted independently the parameters of NMDAR- and eCB-dependent mechanisms using the data of [39, 40] and adding the constraint that for large |Δ*t*| (> 40 ms) no plasticity occurs. These parameters were used to calculate the total change in macroscopic synaptic strength, using the analytical formula, and we depict the result of this calculation as a function of Δ*t* and the number of pairings in Fig 3(a). This calculation allows one to uncover the continuous profile of the synaptic strength change as a function of the number of pairing presentations, and we confirmed that the theoretical calculation is in good agreement with simulations of the full nonlinear system for *N* = 1000 synapses, as shown in Fig 3(b)–3(d) (and S2(a) Fig for the full heatmap).

These results also showed a good qualitative agreement with the experimental data. In particular, we found that for Δ*t* < 0, LTP appears rapidly after a few stimulus presentations (Fig 3(b)), disappears as the number of pairings increases (around 25 stimulus presentations, Fig 3(c)), before re-emerging beyond 50 pairings (Fig 3(d)). The fast emergence of eCB-LTP followed by a slower NMDAR-LTP points towards the fact that the potentiation effects of the eCB pathway are significantly larger than those of the NMDAR pathway. Analyzing the quantitative values of the fit parameters (see Table 1), we indeed observe that $\gamma_{e}^{p}$ is approximately 6 times larger than $\gamma_{n}^{p}$. For Δ*t* > 0, LTD progressively establishes, becoming significant around 40 pairings and strengthening in a monotonic way. Typical STDP profiles illustrative of this Fig 3(b)–3(d) for three sample pairing numbers highlighting the presence of three main regimes: single-sided LTP between 10 and 25 pairings (represented here for 13 pairings), no significant plasticity around 40 pairings, and anti-Hebbian STDP at 100 pairings as observed experimentally in [24, 27, 28]. These STDP arise due to the calcium transients generated by a single pairing, and typical configurations of calcium concentrations and thresholds for one given pairing at Δ*t* = ±10 ms in Fig 3(b)–3(d) show how activity-dependent thresholds relate to the changes detailed above in synaptic plasticity.

**Fig 4:**
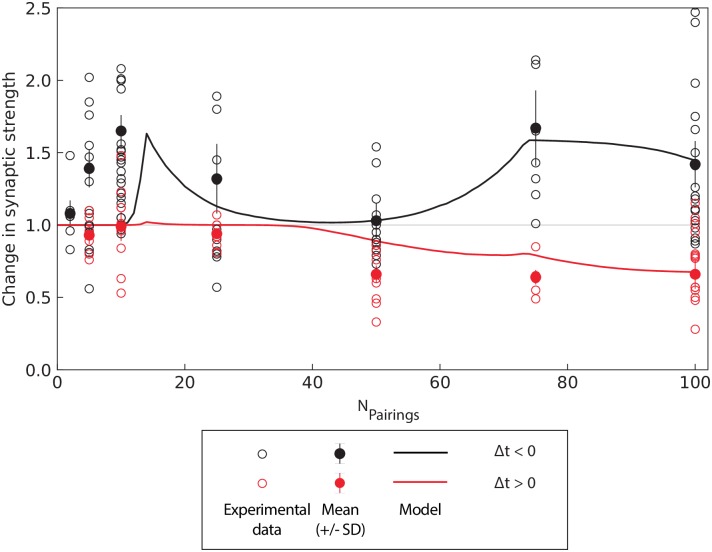
Cortico-striatal model and experimental data. Change in macroscopic synaptic strength (numerical simulations) as a function of the number of pairings in the simplified model show a good agreement with experimental points from [39, 40]. Each experimental point is marked by an empty circle, together with the associated mean (filled circle) and standard deviation (error bar), for pre-post (red) and post-pre (black) pairings. For the model, we performed the mean for +5 ms < Δ*t* < +40 ms and −40 ms < Δ*t* < −5 ms.

The numerical results show a good agreement with the data, as shown in Fig 4. In this figure, we superimposed experimental data points and statistics from [39, 40] together with the numerical simulations of the change in macroscopic synaptic strength for post-pre and pre-post pairings predicted by the model. The only noticeable deviation arises for less than 10 pairings, where the model shows no significant plasticity while experiments show a rapid establishment of plasticity. This may be explained by the rigidity of the bistability hypothesis requiring a significant change in the individual synaptic efficacy to induce a switch from one potential well to the other and, in turn, a variation in the macroscopic synaptic strength.

Finally, we note that our results, derived in the case of piecewise constant thresholds, persist with our more realistic exponential thresholds model (see S2(b) Fig) illustrating the stability of our model and the relevance of piecewise constant approximations of the STDP thresholds.

#### Pathway inactivation in the cortico-striatal synapse

**Fig 5:**
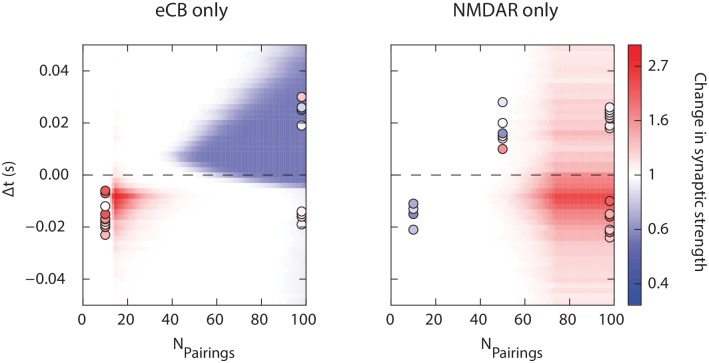
Respective roles of eCB and NMDAR signaling pathways. eCB- (left) and NMDAR- (right) dependent plasticity only computed numerically as a function of the number of pairings and Δ*t*, together with experimental points obtained from [39, 40] by pharmalogically impairing, respectively, NMDAR- or eCB-dependent plasticities.

To test the validity of the cortico-striatal synapse built upon eCB and NMDAR signaling pathways, we computed the plasticity predicted when one of the two mechanisms is inactivated. Experimentally, it was shown that when eCB system was impaired, the synapse displays LTP for post-pre pairings (−30 ms < Δ*t* < 0 ms) arising after high numbers of stimulus presentations (*N*_*Pairings*_ > 70). Blocking NMDAR pathways leads to post-pre LTP for low numbers of pairings progressively disappearing as pairings are presented, and a pre-post LTD (Δ*t* > 0) for large numbers of pairings (> 75) [40]. In our model, blocking eCB pathways is equivalent to computing the synaptic changes through *ρ*_*n*_ only, and blocking NMDAR-dependent pathways reduces synaptic changes to *ρ*_*e*_ only. In these two cases, we obtained a very good agreement of the model simulations with these observations, as shown in Fig 5. We note in our model the emergence of a weak NMDAR-LTP on the pre-post side for large numbers of presentations. This is inconsistent with the experimental data, and related to our choice of considering no NMDAR-LTD to keep a minimal model with a limited set of free parameters reproducing the most prominent aspects of the cortico-striatal STDP. This effect could be readily avoided by adding NMDAR-LTD.

Overall, the model appears thus not only consistent with experimental data on the combined effect of multiple pathways to which it was fitted, but also to the response of the synapse in pharmacological situations whereby one of the pathways is blocked, validating the hypothesis that the present dependence on numbers of presentations relies on distinct and independent mechanisms.

#### Influence of the frequency of stimulus presentation

To further test the stability and to harness the predictive power of the model, we considered the changes in macroscopic synaptic strength and its dependence on the pairing frequency. The analytical formula derived in the Methods section is only valid when the pairing frequency is sufficiently small to ensure that the calcium concentration goes back to its baseline between two pairings; at higher frequencies, deriving a similar formula remains possible, but becomes much more intricate because of the interactions between multiple pairings. Indeed, for pairings presented at high frequency, the synaptic change associated to a pairing interacts with the previous and the following repetitions of the stimulation protocol. For instance, an STDP protocol with pre-post pairings (Δ*t* > 0) presented at a frequency *F* will result in a change depending both on Δ*t*, but also to post-pre pairings at Δ*t*′ = −1/*F* + Δ*t* < 0 (with the following stimulation), a negligible effect for small frequencies (when Δ*t*′ is significantly larger than Δ*t*), but becoming prominent for rapid stimuli and tending to overwhelm timing-dependence (when Δ*t*′ and Δ*t* are of the same order of magnitude). As expected, in response to pairings with increasing frequency, a periodic pattern appears (with a period identical to that of the stimulation), and thus the range of values of relevant Δ*t* decreases to the interval (−1/2*F*, 1/2*F*).

**Fig 6:**
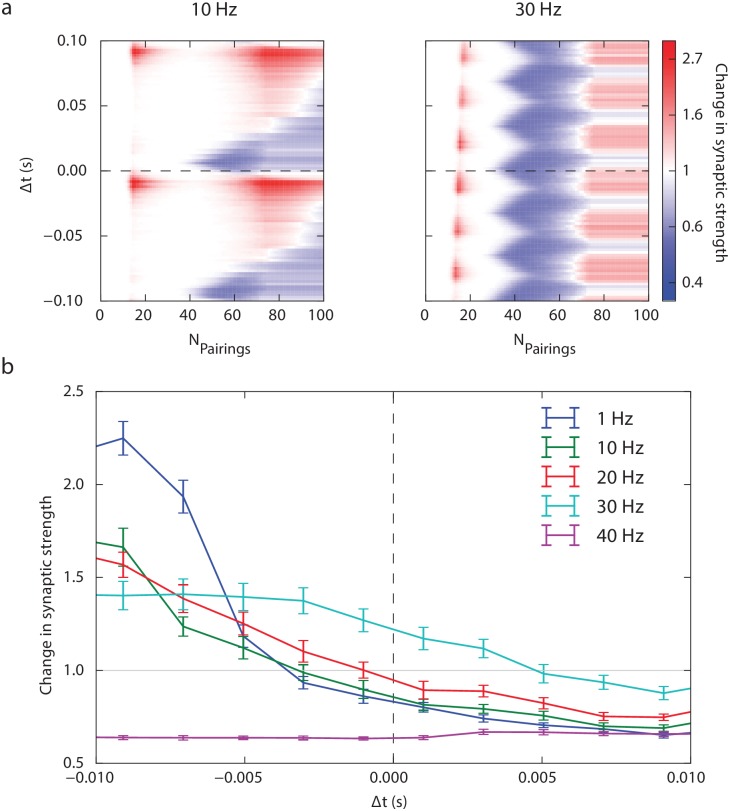
Impact of stimulation frequency. (a) Synaptic strength modification (numerical simulations) as a function of the number of pairings and spike timing Δ*t* when stimuli are presented at 10 Hz and 30 Hz. (b) Average (and standard deviation) of the synaptic strength modification as a function of Δ*t* for 100 stimulus presentations and various frequencies.

Fig 6 represents the response of the model for STDP protocols with various stimulation frequencies. We first observed for 10 Hz and 30 Hz (Fig 6(a)), the appearance of periodic patterns as predicted above. At 10 Hz, the STDP profile seems not to be different (except for the periodic pattern) from the one at 1 Hz. We observed that increasing pairing frequency altered the plasticity profile in several ways. At higher frequencies (30 Hz), plasticity tends to lose its dependence in the spike timing. At 30 Hz pairing frequency and 100 pairings, LTP tends to dominate since LTD has disappeared on the pre-post side and LTP starts emerging. This is illustrated in Fig 6(b) where is depicted a plasticity occurring at 100 pairings for a small range of Δ*t*, highlighting the progressive establishment of a unidirectional LTP as frequency increases. This model prediction is qualitatively consistent with experimental observations [17]. At 30 Hz, the model predicts that an LTD independent of the spike timing (*i.e.* rate-based LTD) should appear between 40 and 60 pairings. From the model viewpoint, this is due to an almost flat unidirectional LTD arising in this range of number of pairings, but to date this remains an open question and requires experimental validation.

A limit of the present model is reached when the frequency is sufficiently high for the calcium trace to remain above LTP and LTD thresholds during the whole stimulation procedure. Depending on the relative values of the LTP and LTD thresholds, either LTP or LTD ends up dominating at high frequency. For higher frequencies (> 55 Hz) in our case, the calcium concentration remained above LTP and LTD thresholds after a small transition period. In this regime, the resulting STDP chiefly relies on the choice of parameters LTD^⋆^ and LTP^⋆^. This effect can be seen for 40 Hz Fig 6(b), where we have a LTD independent of the timing of the spikes. We limited our study up to 30 Hz pairing frequency since for higher frequency our model starts losing biological relevance.

#### Triplets of spikes

Experimental evidence, as well as the model’s predictions, suggest that the presence of multiple pathways induces a dependence of resulting plasticity on the number of stimuli presented. The model allows to predict those dependences. In this section, we characterize the predictions the model provides regarding plasticities induced by triplets of spikes. Such plasticities, arising after repeated presentations of two presynaptic spikes and one postsynaptic spike or conversely, were described experimentally in [38, 55, 58], but to date no experiment has shown the dependence of those *triplet rules* upon variations of the number of stimulus presentations.

Several models of triplets have been developed recently, aiming at reproducing the interaction of three (or more) spikes. In particular, a model of triplets grounded on plasticity data from pyramidal cells of layer 2/3 of rat visual cortex was developed and captured the influence of the pre- and postsynaptic spikes preceding a paired stimulation [38](60-80 stimulus presentations). Another model of interactions between spikes is developped in [59] to reproduce hippocampal data from [58] at 60 pairings. In [60], voltage-based rules of STDP are used to study triplets of spikes at 60 pairings specifically. Simulations with triplets were also performed for a calcium-based model in [47], for two different number of pairings (30 and 100). Finally, a model based on NMDAR kinetics [61] was developed in [37] to study the stability of the weight distributions through numerical simulation of a population of synapses connected to one neuron. All these models focus on Hebbian plasticity and do not include long time influence of the inactivation of the different signaling pathways. The models proposed in [59, 60] considered a fixed number of pairings, except [47] where simulations with two different numbers of pairings (30 and 100) showed a monotonic appearance of the plasticities even for triplets protocols.

**Fig 7:**
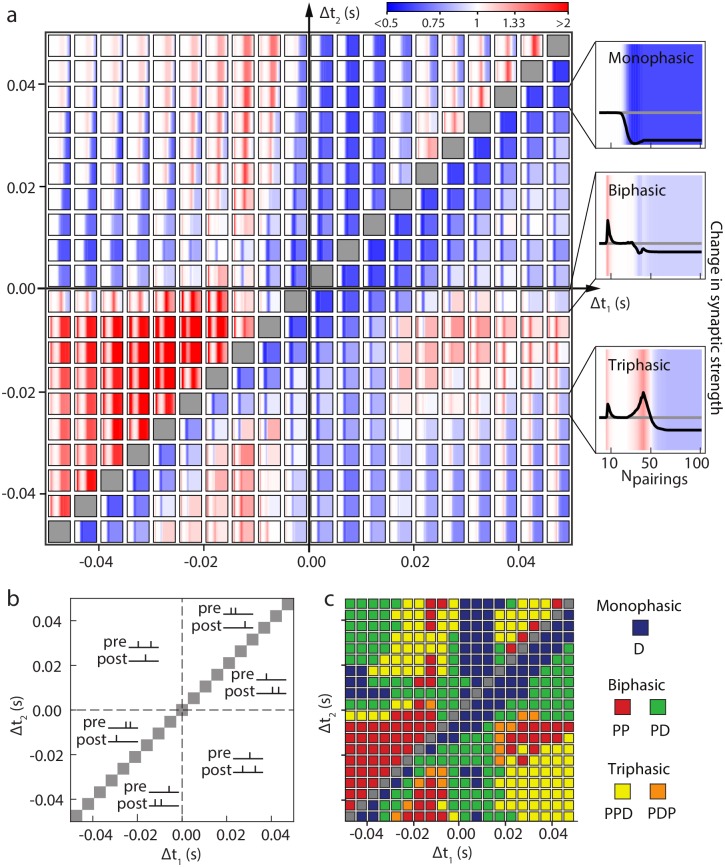
Predictions of the dependence of triplet rules upon numbers of pairings. (a) Synaptic strength modification (numerical simulations) as a function of the number of pairings for different triplets protocols. Each square represents the change in synaptic strength (which intensity is color coded) as a function of the number of pairings, for a precise Δ*t*_1_ and Δ*t*_2_. Insets (right) provide an alternative representation of the same quantity in the monophasic D (up), biphasic PD (middle) and triphasic PPD (down) case. **(b)** Schematic representation of the different triplets configurations in (a) with Δ*t*_*i*_ = *t*_*post*_ − *t*_*pre,i*_ for pre-post-pre stimulations and Δ*t*_*i*_ = *t*_*post,i*_ − *t*_*pre*_ for post-pre-post stimulations. **(c)** Different phases (mono-, bi- and triphasic) of STDP observed in protocols of triplets as a function of Δ*t*_1_ and Δ*t*_2_. P and D code for potentiation (LTP) and depression (LTD).

Our model, incorporating a long-time dependence of inactivation thresholds, highlights new patterns of STDP that strongly depend on the number of triplet presentations. In Fig 7(a) are depicted the changes in synaptic strength as a function of (i) the type of stimulus (number of spikes of the pre- or postsynaptic neuron), (ii) the timing between spikes and (iii) the number of stimulations. Stimulations with two pre- and one postsynaptic spikes are depicted in the upper-left triangle as a function of the timing of the presynaptic spikes relative to the postsynaptic spike Δ*t*_1_ < Δ*t*_2_, and those with one pre- and two postsynaptic spikes are depicted in the lower-right triangle as a function of the timing of the postsynaptic spikes relative to the presynaptic spike, with Δ*t*_1_ > Δ*t*_2_ (see Fig 7(b)). In Fig 7(a), for each value of Δ*t*_1_ and Δ*t*_2_, a heatmap describes the change in synaptic strength as a function of the number of pairings in the case of the cortico-striatal synapse studied above. This diagram shows complex non-monotonic dependences of plasticity as a function of the number of pairings, with vastly distinct profiles depending on the timing.

Indeed, only a small portion of the diagram shows a monophasic establishment of plasticity with only LTD (20%, blue squares in Fig 7(c)), as it would be the case with a single STDP mechanism involved (note that, even for those stimuli, the establishment of plasticity may not be monotonic, as shown in the monophasic inset). The majority of the stimulations therefore yield a non-monotonic establishment of STDP as a function of the number of presentations. We distinguished four main profiles of plasticity establishment:

- biphasic potentiation-depression plasticity (PD), characterized by an early potentiation of the synapse followed by depression (green squares in Fig 7(c), arising for ∼32% of the stimuli considered), characterized by a phase of early eCB-LTP followed by the establishment of a stable eCB-LTD;
- biphasic potentiation-potentiation plasticity (PP), characterized again by an early eCB-LTP followed by an absence of plasticity and the re-emergence of a potentiation relying on NMDAR pathways as stimuli are presented (red squares in Fig 7(c), arising for 20% of the stimuli considered);
- triphasic potentiation-potentiation-depression plasticity (PPD), (yellow squares in Fig 7(c), arising for ∼24% of the stimuli considered), distinct from biphasic PD cases with an early establishment of eCB-LTP, followed by the emergence of an NMDAR-LTP eventually overcome by eCB-LTD;
- triphasic potentiation-depression-potentiation plasticity (PDP), (orange squares in Fig 7(c)), arising only for a few stimulus patterns (∼4% of the stimuli considered), corresponding to an early eCB-LTP disappearing progressively, leaving room for eCB-LTD eventually overcome by NMDAR-LTP (this tight competition between eCB-LTD and NMDAR-LTP explains the small range of parameters where this occurs).

**Fig 8:**
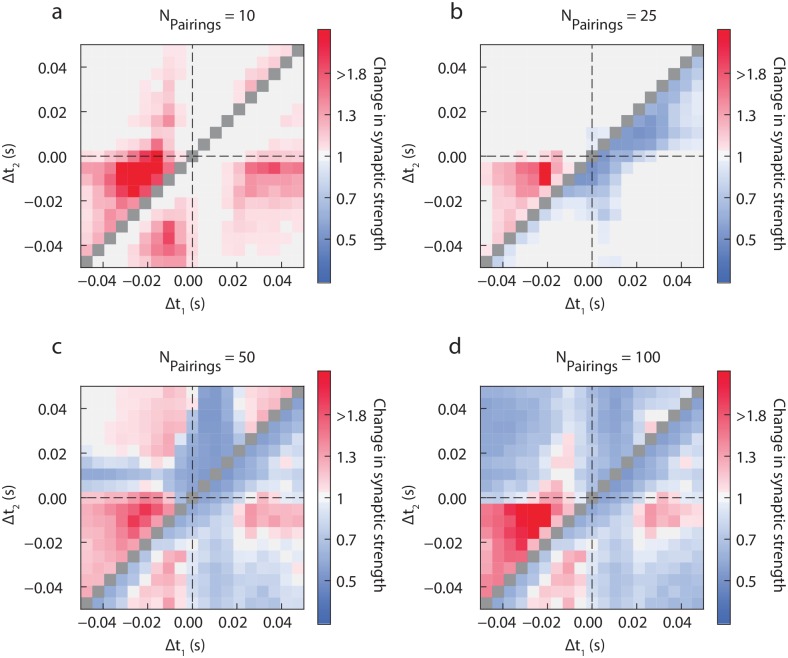
Triplet rules (numerical simulations) for 10, 25, 50 and 100 pairings. (same convention as in Fig 7(b)).

Therefore, the dependence upon the number of triplet presentations highlights the complex interplay of the multiple pathways in the establishment of plasticity for stimuli more complex than spike pairs. In particular, Fig 7 shows that vastly distinct STDPs emerge for fixed numbers of stimulus presentations. We observed that the map of synaptic efficacy changes depends on the number of stimulus presentations Fig 8.

Because of the early activation of eCB-LTP, we observe at low numbers of triplet presentations (see Fig 8(a)) that only LTP is expressed, and is particularly prominent for post-pre-pre presentations (upper-left triangle with Δ*t*_1_ < 0 and Δ*t*_2_ < 0), and smaller plasticity regions for post-post-pre and post-pre-post triplets with respectively Δ*t*_2_ sufficiently negative or Δ*t*_1_ sufficiently large. This LTP relies on the eCB pathway only, as shown in the S3 Fig. The latter plasticities can actually be understood from spike pairing: indeed, when one of the timings Δ*t*_1_ or Δ*t*_2_ is sufficiently large, the synaptic change follows spike pair rules, leading to the observed early eCB-LTP. Similarly, this effect is enhanced when a doublet of postsynaptic spikes precedes a presynaptic spike. At 25 presentations of spike doublets, we have seen in Fig 3 that no significant LTD was present, and only eCB-LTP was expressed. This is not the case for spike triplets. Indeed, triplets have the ability to reveal weak eCB-LTD (Fig 8(b)), particularly in the pre-post-post regime where the doublet of postsynaptic spikes increases the associated calcium peak and leads to the expression of the eCB-LTD significantly earlier than for spike pairs. eCB-LTP persists for post-pre-pre stimulations, but no significant LTP was found for post-pre-post or pre-post-post stimulations, for which the calcium spike does not exceed the increased threshold anymore due to past calcium transients. For 40 triplets (not shown), the eCB-LTP in the post-pre-pre regime is significantly reduced, and eCB-LTD influence broadens, while NMDAR-LTP emerges in the post-post-pre, post-pre-post and pre-pre-post regions, significantly earlier than the NMDAR-LTP arising for doublets of spikes. This emergence is even more visible for 50 triplet presentations (Fig 8(c)), together with the appearance of the NMDAR-LTP in the post-pre-pre region. A new pocket of LTP arises also in the pre-post-pre region with Δ*t*_1_ < Δ*t*_2_, where the NMDAR-LTP induced by the post-pre pairings of spikes overcomes the eCB-LTD of the pre-post pairings of spikes. Interestingly, this new regime disappears as the number of stimulus presentations is increased, arising together with the full expression of eCB-LTD for 100 pairings (Fig 8(d)). The post-pre-pre NMDAR-LTP reaches larger values than in the case of doublets at 100 pairings Fig 8(d). A movie of the variation of synaptic efficacy as a function of the number of pairings is provided in Supplementary Movie M1.

### Variety and diversity of plasticity rules with multiple signaling pathways

STDP at the cortico-striatal synapse, studied in the previous section, provides a realistic example of plasticity with multiple pathways. Our model, relying on only two equations and a small number of biologically interpretable parameters emulating NMDAR- and eCB-dependent pathways, reproduces all the phenomena reported at the cortico-striatal synapse, and allowed to draw predictions on plasticity for more complex stimuli such as triplet rules. The present model is however much more general than the case of the cortico-striatal synapse: it can indeed emulate synapses with more than two signaling pathways with arbitrary independent plasticity rules, and thus allows unraveling the dynamics of plasticity expression in a variety of synapses with distinct plasticity. Interestingly, while being quite versatile, the repertoire of behaviors that can be reproduced given a fixed number of pathways remains limited, and the model thus also provides predictions on the minimal number of pathways involved given a plasticity profile. Indeed, a single pathway shall induce a monotonic establishment of plasticity if there is no inactivation of the pathway, whereas situations with two pathways can lead to four changes of plasticity (LTP and LTD inactivation for each of the two pathways), possibly with periods of non-significant synaptic changes. More generally, plasticity with *P* pathways may lead to up to 2*P* changes of monotonicity, possibly interspersed with periods of non-significant plasticity.

We investigate in the next sections a few possible scenarii relying on at most two signaling pathways that could lead to Hebbian or anti-Hebbian plasticity and suggest experiments that could distinguish distinct situations.

#### Symmetric anti-Hebbian LTD

In neocortical excitatory synapses onto inhibitory interneurons, 60 pairings at 1 Hz leads to an anti-Hebbian symmetric LTD [32]. Another example of anti-Hebbian symmetric LTD has been found at the synapses between parallel fibers and Purkinje-like cells of the electrosensory lobe of mormyrid electric fish for a protocol of 60 pairings at 0.5 Hz [34]. It remains unknown how the establishment of this plasticity depends on the number of presentations of the stimulus or on the frequency of presentations, and those can have dramatic effects if multiple pathways contribute to this phenomenon as in the case of the cortico-striatal synapses. Indeed, anti-Hebbian LTD can unfold into diverse profiles as a function of the number of pairings, and we explore a part of this diversity here, limiting our exploration to the simplest non-trivial case of plasticities relying on up to two signaling pathways. We focus on three possible scenarii constrained to reproduce symmetric LTD at 100 pairings and 1 Hz.

**Fig 9:**
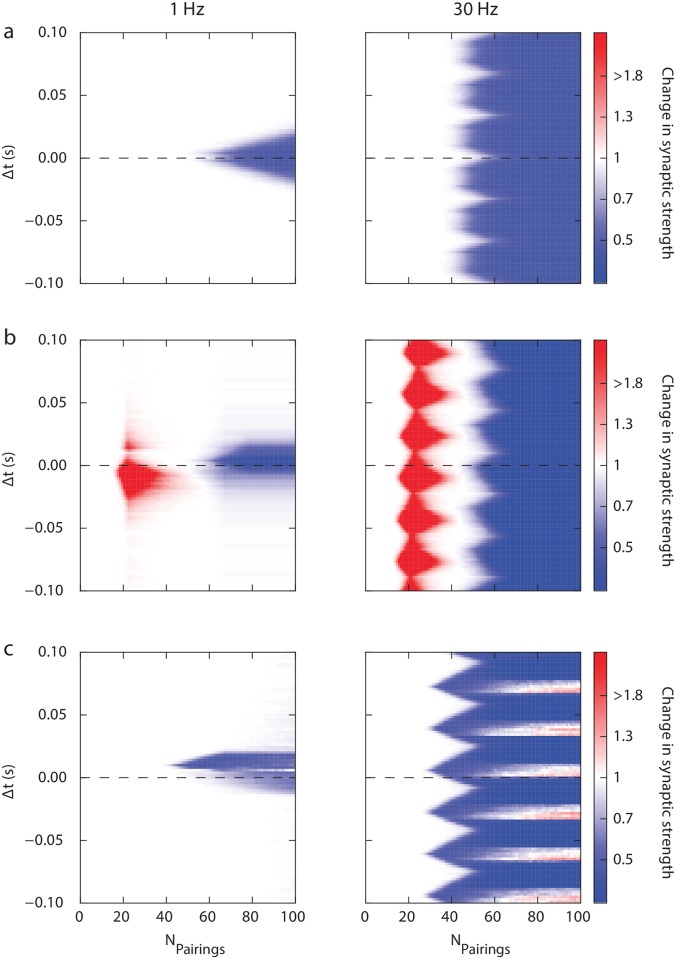
Distinct mechanisms leading to unidirectional LTD at 100 pairings and 1 Hz. Synaptic strength modification (numerical simulations) as a function of the number of pairings and Δ*t* for a synapse **(a)** with a single plasticity mechanism (**Scenario 1**), **(b)** relying on two mechanisms, one of which inducing an early LTP inactivating as the number of pairings increases (**Scenario 2**), or **(c)** relying on two mechanisms, one of which inducing a Hebbian STDP and the second inducing a pre-post LTD (**Scenario 3**).

**Scenario 1**: The simplest plasticity framework leading to symmetric LTD is composed of a single plasticity pathway. In that case, LTD establishes monotonically without LTP expression (see Fig 9(a) and parameters in Table 1). Variations in the frequency of the stimulus presentation does not reveal any potentiation, and raising frequency up to 30 Hz leads to a constant LTD independent of spike timings.

The situation is more complex when two signaling pathways contribute to the establishment of the symmetric anti-Hebbian plasticity. Multiple scenarii can be designed leading to unidirectional LTD at 100 pairings at 1 Hz. Here, we consider situations where two pathways interact, one of which (pathway A) induces LTD and LTP at a prescribed number of stimulus presentation, and the other mechanism (pathway B) only leading to LTD:

**Scenario 2**: Pathway A leads to an early emergence of LTP that inactivates as pairing numbers are increased. This mechanism is similar to the case of eCB-LTP in the cortico-striatal synapse studied above.
**Scenario 3**: Pathway A leads to an enduring LTP not inactivated when the number of pairings is increased, but which is dominated by the LTD generated by pathway B.

In detail, we consider in **Scenario 2** that pathway A generates an early post-pre LTP that inactivates as the number of pairings increases, and a late-appearing pre-post LTD. At the same time, pathway B generates a unidirectional LTD arising after the early LTP of pathway A (see S4(a) Fig depicting individual plasticity profiles for each mechanism when the other is inhibited, and parameters in Table 1). In this situation, LTP disappears as pairing numbers increase, and LTD takes over leading to bilateral LTD at 100 pairings (see Fig 9(b)). When presenting stimuli at higher frequency, timing-dependence disappears in favor of a constant LTD at 100 pairings, and thus experiments based on the presentation of a fixed and large number of pairings will not allow to distinguish **Scenario 1** from **Scenario 2**. However, the response of the synapse in **Scenario 1** and **Scenario 2** as a function of the number of pairings at 30 Hz is distinct. Particularly, at 20 pairings, **Scenario 2** leads to a constant bilateral LTP, vastly distinct from the absence of plasticity arising at this frequency and numbers of pairings in the single-mechanism case.

In **Scenario 3**, the plasticity pathway B leads to a unilateral pre-post LTD, while pathway A induces a Hebbian STDP at 100 pairings and 1 Hz (LTP for Δ*t* > 0, LTD for Δ*t* < 0, see S4(b) Fig depicting the plasticity induced by each pathway independently, and Table 1 for parameters). When the LTD of mechanism *A* dominates the LTP of mechanism *B*, a unidirectional LTD was observed at 100 pairings (1 Hz), and no significant region of LTP arises at this frequency (see Fig 9(c)). However, increasing the pairing frequency yields vastly distinct results, and the underlying LTP, invisible for presentations of the stimuli at lower frequencies, re-emerges significantly for pre-post pairings, and the spike-timing dependence of plasticity strengthens and displays a Hebbian STDP profile locally.

These STDP examples show a novel phenomenon: multiple independent mechanisms not only affect emergent long-term plasticity for low number of pairings, but they can also lead to non-trivial emergent plasticities when stimulation protocols are modified. These phenomena cannot be predicted from the observation of the result of stimulation protocols with a fixed and large number of pairings presented at low frequency.

#### Hebbian plasticity

We chose to examine in the previous sections two types of anti-Hebbian STDPs, asymmetric STDP [27, 28] and symmetric LTD [32, 34]. The model is also able to reproduce the whole spectrum of plasticities described in [8], in the flavor of the simulations performed in [47]. Asymmetric Hebbian plasticity is commonly observed at various synapses [11–23, 62], and can be readily studied along the same lines as the anti-Hebbian case described here, inverting LTP and LTD. Hebbian symmetric plasiticty is observed in the hippocampus [36]. We show for instance in S1 Fig the case of Hebbian symmetric or asymmetric STDP using parameters taken from [47], supported by a single plasticity pathway. Our present simplified mathematical model is thus able to reproduce and predict various forms of STDP at play in neural circuits.

## Discussion

Synaptic plasticity is a complex phenomenon relying on the activation of a number of receptors and signaling pathways [3, 10]. A substantial difficulty for experimentalists is to characterize plasticity in the large variety of possible situations occurring *in vivo*. To reduce this complexity, a protocol designed to reveal plasticity consists in considering changes in synaptic transmission after the reiterated presentation of a fixed spike pattern a large number of times (on the order of one hundred) and at a slow rate. From these experiments, it remains complex to decipher the multiple signaling pathways involved in the expression of plasticity, and their complex interplay, particularly for low numbers of stimulus presentations or for various pairing frequencies.

To disentangle the distinctive role of multiple pathways, we developed and studied a phenomenological model of the evolution of synaptic weights and tested its responses in distinct situations. The model relies on calcium transients triggered by the spiking activity of neurons on both sides of the synapses, and is built upon previous theoretical works (see [47] and references therein). When plasticity (LTP and LTD) relies on multiple signaling pathways [10], the timescales at which these mechanisms activate and inactivate upon repetitive stimulation can lead to a variety of behaviors as a function of the number and of the frequency of pairings, which cannot be inferred from experiments where those are fixed. Our model proposes a general and minimal framework to integrate multiple signaling pathways and their dependences upon repetitive stimulations. We have instantiated this model with two specific pathways, NMDAR- and eCB-dependent, that was inspired by experiments at cortico-striatal synapse showing variations of the emergent plasticity upon variation of the number of pairings [39, 40]. Our model reduces to two stochastic equations Eq (6) and a small number of parameters, and accurately reproduced the data obtained in that experimental contribution. To our knowledge, this model is the most parsimonious model reproducing STDP experimental results, yet many models of the class that we introduced can be proposed, including for instance NMDAR-LTD or pathways activated by distinct molecules. We also used the model to predict the response of the system when the number of stimulations, the pairing frequency or the number of spikes, are varied. This led us to draw predictions on the modifications of STDP profiles when the frequency of stimulus presentations was varied. Eventually, we have made new predictions on the dependence of triplet rules upon the number of stimulus presentations, and showed that complex non-monotonic STDP profiles emerge with up to three distinct phases. Our model goes beyond the particular case of the cortico-striatal synapse for which data was available, and we pursued our investigations by considering distinct mechanisms that could underlie another type of plasticity, symmetric anti-Hebbian LTD (with LTD for pre-post and post-pre pairings). In this case, we investigated three distinct possible scenarii involving up to two distinct pathways, and showed that unexpected phenomena may arise upon variations of the number and frequency of pairings, and in particular the emergence of an LTP at 100 pairings for high frequencies. Overall, these results highlight the fact that electrophysiological experiments at a fixed frequency and a prescribed number of pairings may not be sufficient to extrapolate to other situations with smaller numbers of pairings or presentation frequencies.

To our knowledge, the present model is the first to take into account distinct signaling mechanisms involved in plasticity in a simple and compact framework. The simplicity of the present model allows to envision the implementation of this type of synapse at the level of a neural network, opening the way to theoretical studies of information processing capacity of networks endowed with complex activity-dependent plasticity rules. In addition to the development of a framework integrating multiple pathways, one of the main novelties of this model compared to other calcium-based models is that we have explicitly incorporated activity-dependent thresholds allowing to recover the response of plasticity mechanisms on the past activity of cells. In the present model, we simply assumed that this history-dependence is parameterized by a cumulative calcium concentration. Explicitly incorporating this dependence allows taking into account in the model multifarious experimental facts including finiteness of the calcium pool in the postsynaptic compartment, desensitization of synaptic-receptors and homeostasic mechanisms [63]. The present model proposing that this dependence on past activity relies on cumulative calcium constitutes a first step, and could be refined in several directions, for instance incorporating a slow decay of past-activity dependence with time (considering integrated calcium spikes with an exponentially decaying kernel for instance), moving averages in the flavor of sliding thresholds in the classical Bienenstock-Cooper-Munro (BCM) rule [56, 57]. In our case, the average activity of the neuron would be simply modeled by postsynaptic calcium concentration (a reasonable proxy of neural activity), or with more refined models involving distinct molecular species and their timescales.

Despite a good qualitative agreement and an improved accuracy on the dynamics of the expression of plasticity, we found that our model shows a slight mismatch in the timescales at which plasticity emerges: first, although experiments at cortico-striatal synapses show a significant plasticity arising as early as 5 pairings and reaching a maximum at 10 pairings, we did not find in the model significant plasticity at 5 pairings and the maximal plasticity occurred after a slightly larger number of 12 pairings. Moreover, a unidirectional LTP in the cortico-striatal plasticity at 100 pairings was observed experimentally when the frequency of pairing presentations reached 4 Hz, while the model reproduces this phenomenon slightly above 30 Hz. We believe that this slower response of the present model relies on the bistable nature of the model, following [47]. This bistability makes the system quite rigid and resistant to rapid changes, and a direct perspective would be to implement a more flexible model dropping the bistable model but conserving the long-term stability of macroscopic synaptic strength ensured by the bistable potential. The present model would be also used in future works focusing on the implementation of the cortico-striatal STDP in large stochastic neural networks, with several classes of interneurons, aimed at understanding the possible role of implementing distinct cortico-striatal plasticity, in particular LTP, arising at various timescales and their possible role in information processing in striatum.

All in all, the present model suggests to reconsider a current widely admitted implicit hypothesis in models, and questions the usual view of STDP in models that consider a fixed curve solely dependent on the spike timing (Δ*t*). Indeed, in most neural network models with STDP, it is considered that synaptic coefficients are progressively incremented depending on spike timings and according to toy-models of STDP (e.g., double-exponential curves). This is implemented in various manners, including additive or multiplicative changes depending on all spike pairings or on the nearest-spike (see e.g. [64]).

At the level of networks, a number of stochastic models were developed to study the influence of STDP as a synaptic plasticity rule (see the review [4]). In particular, early works showed the influence of classical Hebbian and asymmetric STDP in the dynamics of neuronal networks [65–67]. The role of STDP-based rules in the emergence of structures in recurrent neural networks was also studied in a series of papers, highlighting for instance a possible key impact on the self-organization of microcircuits [68–70]. More recently, the distribution of synaptic weights and its stability in randomly stimulated networks with different triplet rules has been extensively studied [37]. The activity-dependent rule we proposed here, reproducing variable synaptic changes as a function of the number of stimulations, may lead to significant changes in the resulting connectivity and dynamics of neural networks. Our model offers an avenue to revaluate the possible modifications of the resulting dynamics emphasizing the role of timescales in these systems [71].

## Methods

### Numerical methods and parameters

Simulations were performed with a custom code implemented in Python 2.7 or 3.5, developed within the *Spyder* environment of the *Anaconda* suite (*Anaconda Software Distribution*. Computer software. Vers. 2-2.4.0. Continuum Analytics, Nov. 2015. Web. <https://continuum.io>). The main modules used *numpy*, *matplotlib*, *math*, *scipy*. Elementary simulations of the model were run on a Macbook Pro (Intel Core i5 processor and 16 RAM) and more demanding simulations were executed on the Inria Paris-Rocquencourt computer cluster. Figures and plots were realized using *matplotlib* module of Python and Illustrator/Photoshop of the Adobe series.

Unless specified otherwise, we used the parameter values listed in Table 1. These parameters were optimized starting from initial guesses chosen for consistency of the model with the data using the extensive analysis of [47, Fig 2]. For adjusting our parameters to the cortico-striatal plasticity, we used a global optimization algorithm, the *differential-evolution* function from *scipy.optimize* module to obtain qualitative fits.

Simulations of the model were realized either from the theoretical expressions computed, or with numerical simulations of the system of stochastic equations Eq (6). We used temporal discretization using an Euler scheme on *t* = −1…101 s with *N*_*iter*_ steps (see Table 1) and run the simulation for a set of *N* = 1000 individual efficacies. To compute the change in macroscopic synaptic strength for the different pairings, we run the simulation for *N*_*Pairings*_ = 100 pairings and store the results for all the pairings during the STDP protocol. Therefore, for each fixed Δ*t*, the results obtained for different pairings are not independent, which has the interest of uncovering the evolution of one given synapse, and has no impact on the global outcome of the simulations as can be seen when compared with analytical results. Except for Fig 3 where the analytical mean-field solutions are represented, all the figures show the numerical simulations.

For Fig 6, we have reproduced 30 independent simulations in parallel to obtain the statistical means and standard deviations depicted. All heatmaps used a logarithmic color bar to represent changes in synaptic strength. The classification in mono-, bi- or tri-phasic regimes in Fig 7(c) was done through a visual inspection of the STDP curves associated to each of the 20 Δ*t*_1_ and Δ*t*_2_.

### The Ornstein-Uhlenbeck approximation

The model we studied is nonlinear, and as such, it was complex to derive the explicit form of the probability distribution of the solutions. Following the approach proposed in the Appendix of [47], we derived the probability distribution of the solution of an approximate model valid when the system remains in the linear part of the cubic bistable term. The model involves a linear Ornstein-Uhlenbeck with deterministic time-dependent coefficients *α*(*t*) and *β*(*t*) that we computed as follow. The solution of linear stochastic differential equations of type [72] (with *B* a standard Brownian motion):

$$\begin{array}{c} d\rho \left(t\right)=(\alpha (t)\rho \left(t\right)+\beta (t))dt+\sigma (t)dB(t) \end{array}$$ (7)

with initial condition *ρ*_0_ can be easily expressed in closed form as:

$$\begin{array}{c} \rho \left(t\right)=\rho_{0}\exp(\int_{0}^{t}\alpha (s)ds)+\int_{0}^{t}\exp(\int_{u}^{t}\alpha (s)ds)\beta (u)du\\ +\int_{0}^{t}\exp(\int_{u}^{t}\alpha (s)ds)\sigma (u)dB(u). \end{array}$$ (8)

As indicated in the main text, the synaptic change is obtained using a sigmoid transform of the proportion *U* of synapses that, after the protocol, have crossed upwards the threshold value ρ*, over the proportion *D* of those that crossed downwards. Since the above described Ornstein-Uhlenbeck process is Gaussian, these probabilities are fully characterized by the mean and single-time variance functions of *ρ*, which have the following expressions:

$$\begin{array}{c} E\left[\rho \left(t\right)|\rho_{0}\right]=\rho_{0}\exp(\int_{0}^{t}\alpha (s)ds)+\int_{0}^{t}\exp(\int_{u}^{t}\alpha (s)ds)\beta (u)du \end{array}$$ (9)

and

$$\begin{array}{c} \text{Var}\left[\rho \left(t\right)|\rho_{0}\right]=\int_{0}^{t}\exp(2\int_{u}^{t}\alpha (s)ds)\sigma^{2}(u)du \end{array}$$ (10)

We thus derive the time-varying coefficients *α* and *β* arising in the approximated model, for the eCB pathway (the other can be dealt with in the same way). These are computed describing the time spent above the various thresholds of the model. We denote by $\eta_{i}^{x}$ the average time spent above the threshold $\theta_{i}^{x}$: this quantity only depends on the calcium dynamics can be easily computed analytically for each given a pairing protocol. Similarly, we define *t*_*e*_ = *T* *n*_*e*_ the time at which the eCB-LTP is inactivated at the cortico-striatal synapse, with *T* being the duration between two pairings and *n*_*e*_ the pairing number at which eCB-LTP is first inactivated.

Denoting $\Gamma_{i}^{x}=\gamma_{i}^{x}\eta_{i}^{x}$, we have the following compact formulae for the coefficients of the Ornstein-Uhlenbeck processes *α*_*e*_, *β*_*e*_ and *σ*_*e*_:

$$\begin{array}{c} \alpha_{e}\left(t\right)=\{\begin{array}{ccc} -\frac{\Gamma_{e}^{d}+\Gamma_{e}^{p}}{\tau }=-\frac{1}{\tau_{1}} & \text{if} & t<t_{e}\\ -\frac{\Gamma_{e}^{d}}{\tau }=-\frac{1}{\tau_{2}} & \text{if} & t_{e}<t\; \end{array} \end{array}$$ (11)

$$\begin{array}{c} \beta_{e}\left(t\right)=\{\begin{array}{ccc} \frac{\Gamma_{e}^{p}}{\tau }=\frac{{\tilde{\rho }}_{1}}{\tau_{1}} & \text{si} & t<t_{e}\\ 0 & \text{si} & t_{e}<t \end{array} \end{array}$$ (12)

$$\begin{array}{c} \sigma_{e}\left(t\right)=\{\begin{array}{ccc} \sqrt{\frac{\eta_{e}^{d}+\eta_{e}^{p}}{\tau }}\sigma =\frac{\sigma_{1}}{\sqrt{\tau_{1}}} & \text{si} & t<t_{e}\\ \sqrt{\frac{\eta_{e}^{d}}{\tau }}\sigma =\frac{\sigma_{2}}{\sqrt{\tau_{2}}} & \text{si} & t_{e}<t \end{array} \end{array}$$ (13)

Because of the simple, piecewise constant form of the coefficients, we have, for deterministic initial conditions:

$$\begin{array}{c} E\left[\rho_{e}\left(t\right)|\rho_{e}\left(0\right)\right]=\{\begin{array}{c} \rho_{e}\left(0\right)\exp(-\frac{t}{\tau_{1}})+{\tilde{\rho }}_{1}(1-\exp(-\frac{t}{\tau_{1}}))\;\text{if}\;t<t_{e}\\ \rho_{e}\left(0\right)\exp(-\frac{t-t_{e}}{\tau_{2}}-\frac{t_{e}}{\tau_{1}})+{\tilde{\rho }}_{1}\exp(-\frac{t-t_{e}}{\tau_{2}})(1-\exp(-\frac{t_{e}}{\tau_{1}}))\\ \;\;\;\;\;\;\;\;\;\;\text{if}\;t_{e}<t \end{array} \end{array}$$ (14)

and

$$\begin{array}{c} \text{Var}\left[\rho_{e}\right]=\{\begin{array}{cc} \sigma_{1}^{2}(1-\exp(-2\frac{t}{\tau_{1}})) & t<t_{e}\\ \sigma_{1}^{2}\exp(-2\frac{t-t_{e}}{\tau_{2}})(1-\exp(-2\frac{t_{e}}{\tau_{1}}))+\sigma_{2}^{2}(1-\exp(-2\frac{t-t_{e}}{\tau_{2}})) & \text{if}\;t_{e}<t \end{array} \end{array}$$ (15)

The probability that an initially depressed synapse becomes potentiated is thus given by:

$$\begin{array}{c} U_{e}\left(nT\right)=P(\rho_{e}>\rho *|\;\rho_{e}\left(0\right)=0)=\frac{1}{2}\left(1+\text{erf}\left(-\frac{\rho *-E[\rho_{e}|\;\rho_{e}\left(0\right)=0]\left(nT\right)}{\sqrt{\text{Var}\left[\rho_{e}\right]\left(nT\right)}}\right)\right), \end{array}$$ (16)

and the probability of an initially potentiated synapse to become depressed by:

$$\begin{array}{c} D_{e}\left(nT\right)=P(\rho_{e}<\rho *|\;\rho_{e}\left(0\right)=1)=\frac{1}{2}\left(1-\text{erf}\left(-\frac{\rho^{*}-E[\rho_{e}|\;\rho_{e}\left(0\right)=1]\left(nT\right)}{\sqrt{\text{Var}\left[\rho_{e}\right]\left(nT\right)}}\right)\right). \end{array}$$ (17)

allowing directly to obtain the change in synaptic weight associated as $H\left(\frac{U_{e}(nT)}{D_{e}(nT)}\right)$.

A comparison of the Ornstein-Uhlenbeck approximation with the numerical simulations of the nonlinear system is provided in Fig 3(a) and S2(a) Fig, showing a good agreement for the parameter set chosen.

### Experimental data points

The data used to fit and validate our results were previously published in [39, 40]. We refer to these papers for more specific information on the experimental protocol.

## Supporting information

S1 Fig Hebbian STDP. Change in the synaptic strength (numerical simulations) as a function of the number of pairings and Δ*t* for asymmetric **(a)** and symmetric **(b)** Hebbian STDP.
(TIF)

S2 Fig Piecewise constant thresholds are a good approximation of exponential thresholds. Change in the synaptic strength (numerical simulations) as a function of the number of pairing and spike timing Δ*t* for **(a)** piecewise constant thresholds or **(b)** exponential thresholds (*ϵ* = 1) show a good qualitative and quantitative agreement.
(TIF)

S3 Fig Role of eCB and NMDAR pathways in triplet rules. Change in synaptic strength (numerical simulations) for the same pairing numbers as in Fig 8 and with the same convention of representation as in Fig 7(b).
(TIF)

S4 Fig Symmetric LTD induction. Change in the synaptic strength (numerical simulations) induced by each individual mechanism in **Scenario 2** **(a)** and **Scenario 3** **(b)** as a function of the number of pairings and spike timing Δ*t*.
(TIF)

S1 Movie STDP varying as a function of the number of pairings in a triplet protocol. Time corresponds to the number of pairings, with a rate of 3 frames per second; the number of pairings is indicated on the top of the graph. Same convention as in Fig 7(b).
(AVI)
