# Supplementary figures and images for: Interplay of multiple pathways and activity-dependent rules in STDP
Source: PLoS Comput Biol. 2018 Aug 14;14(8):e1006184. doi: 10.1371/journal.pcbi.1006184 (PMC6112684; doi:10.1371/journal.pcbi.1006184)

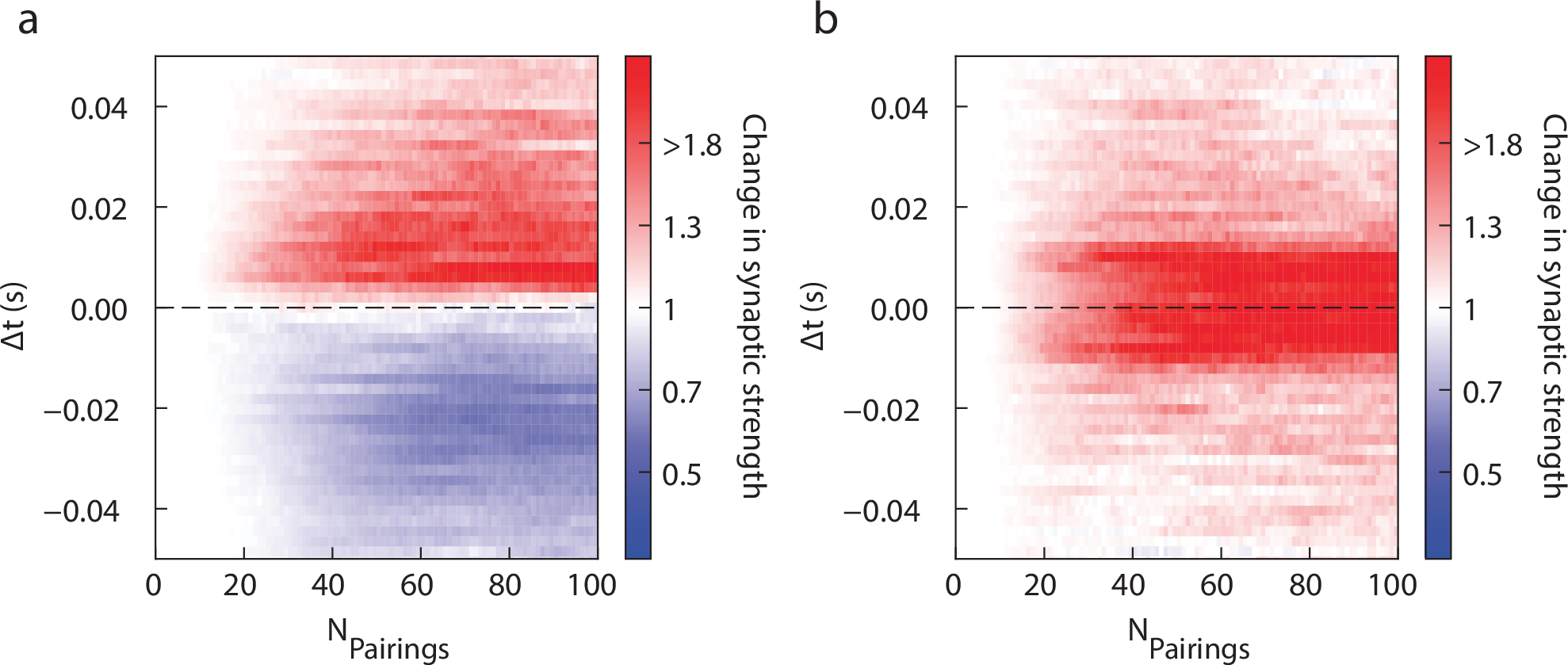

Supplement: S1 Fig — Change in the synaptic strength (numerical simulations) as a function of the number of pairings and Δt for asymmetric (a) and symmetric (b) Hebbian STDP. (TIF) [file pcbi.1006184.s001.tif]

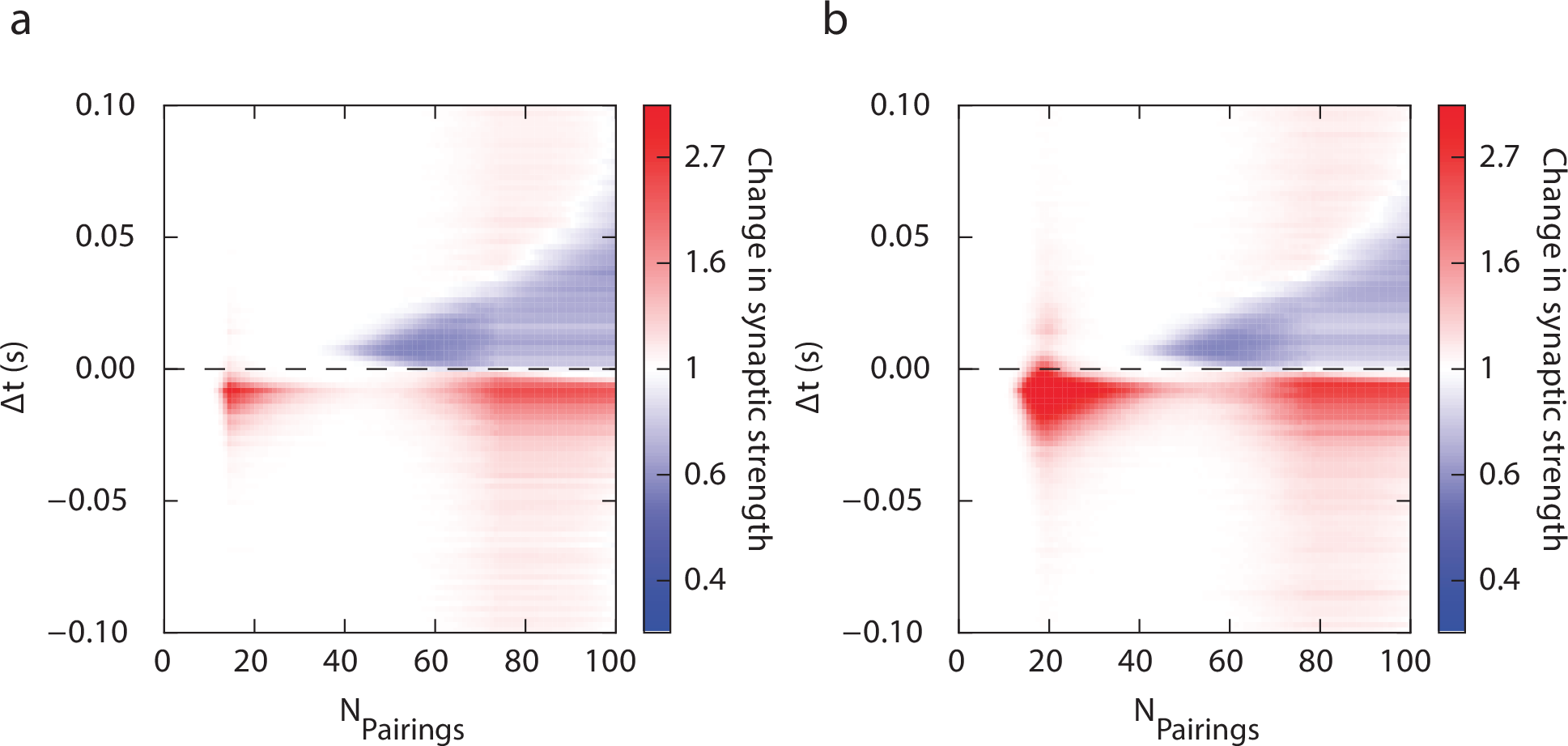

Supplement: S2 Fig — Change in the synaptic strength (numerical simulations) as a function of the number of pairing and spike timing Δt for (a) piecewise constant thresholds or (b) exponential thresholds (ϵ = 1) show a good qualitative and quantitative agreement. (TIF) [file pcbi.1006184.s002.tif]

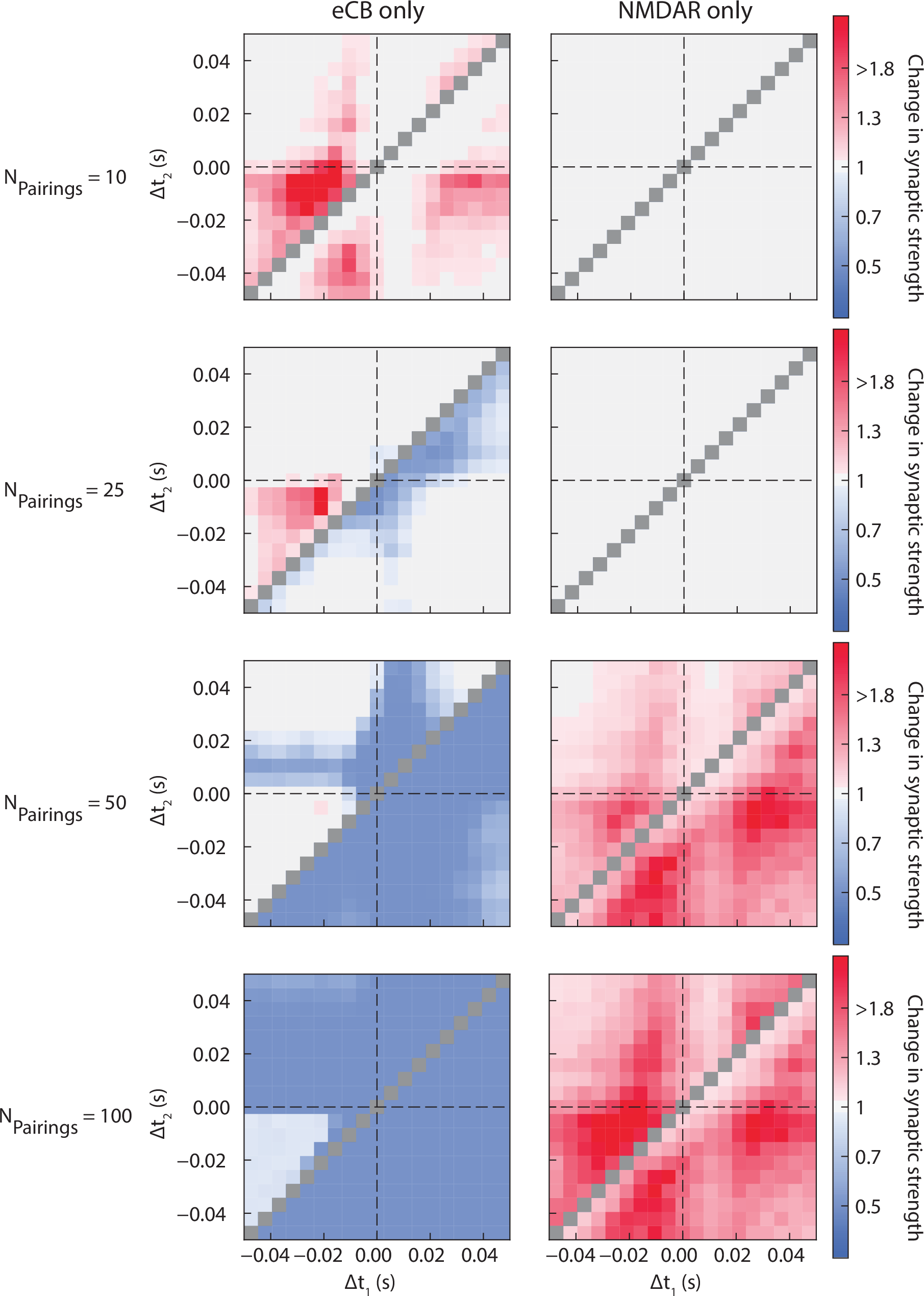

Supplement: S3 Fig — Change in synaptic strength (numerical simulations) for the same pairing numbers as in Fig 8 and with the same convention of representation as in Fig 7(b). (TIF) [file pcbi.1006184.s003.tif]

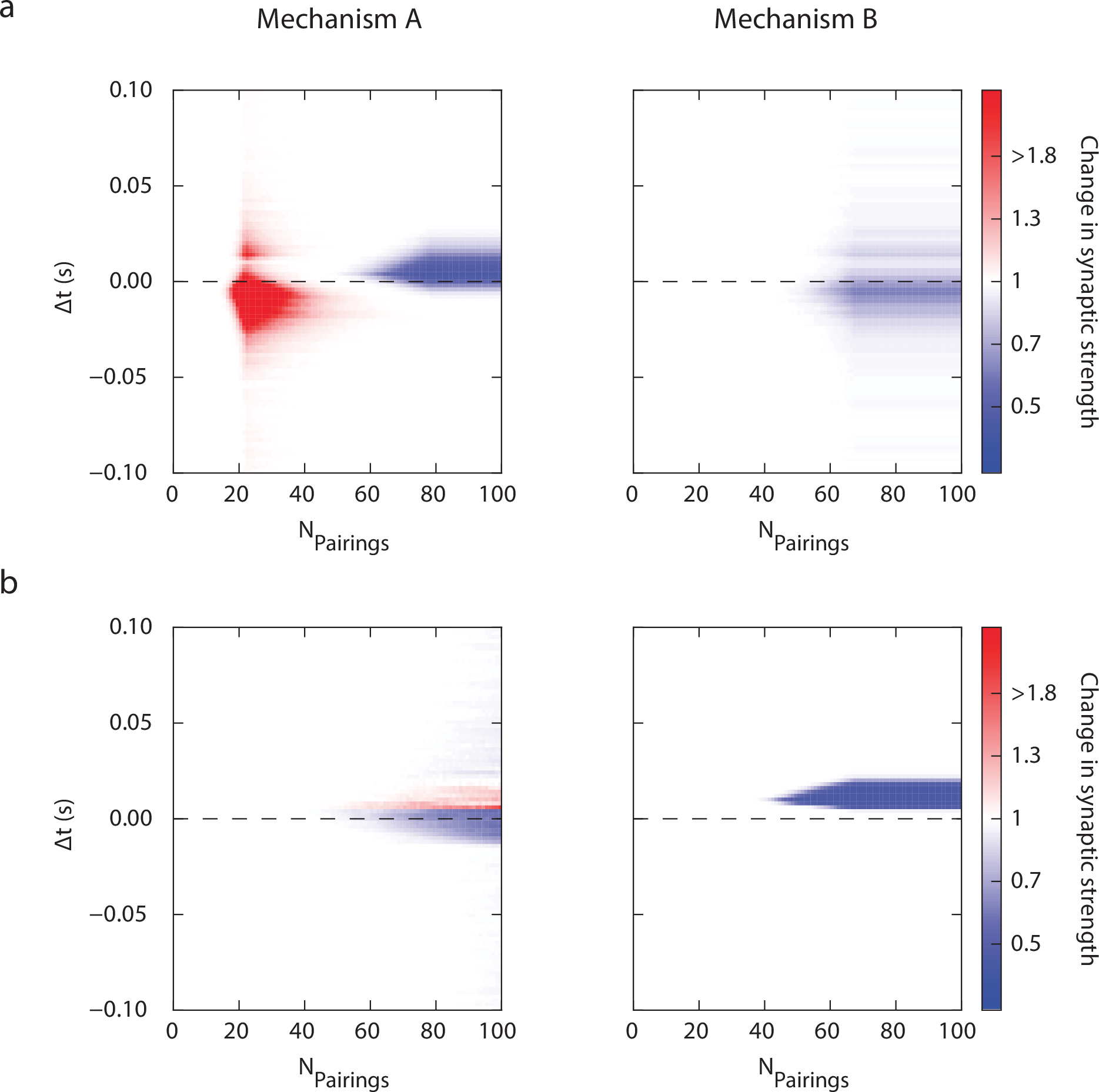

Supplement: S4 Fig — Change in the synaptic strength (numerical simulations) induced by each individual mechanism in Scenario 2 (a) and Scenario 3 (b) as a function of the number of pairings and spike timing Δt. (TIF) [file pcbi.1006184.s004.tif]
